# Regulatory and Metabolic Networks for the Adaptation of *Pseudomonas aeruginosa* Biofilms to Urinary Tract-Like Conditions

**DOI:** 10.1371/journal.pone.0071845

**Published:** 2013-08-13

**Authors:** Petra Tielen, Nathalie Rosin, Ann-Kathrin Meyer, Katrin Dohnt, Isam Haddad, Lothar Jänsch, Johannes Klein, Maike Narten, Claudia Pommerenke, Maurice Scheer, Max Schobert, Dietmar Schomburg, Bernhard Thielen, Dieter Jahn

**Affiliations:** 1 Institute of Microbiology, Technische Universität Braunschweig, Braunschweig, Germany; 2 Institute of Biochemical Engineering, Technische Universität Braunschweig, Braunschweig, Germany; 3 Research Group Cellular Proteomics, Helmholtz Centre for Infection Research, Braunschweig, Germany; 4 Institute of Bioinformatics and Biochemistry, Technische Universität Braunschweig, Braunschweig, Germany; University of Osnabrueck, Germany

## Abstract

Biofilms of the Gram-negative bacterium *Pseudomonas aeruginosa* are one of the major causes of complicated urinary tract infections with detrimental outcome. To develop novel therapeutic strategies the molecular adaption strategies of *P. aeruginosa* biofilms to the conditions of the urinary tract were investigated thoroughly at the systems level using transcriptome, proteome, metabolome and enzyme activity analyses. For this purpose biofilms were grown anaerobically in artificial urine medium (AUM). Obtained data were integrated bioinformatically into gene regulatory and metabolic networks. The dominating response at the transcriptome and proteome level was the adaptation to iron limitation via the broad Fur regulon including 19 sigma factors and up to 80 regulated target genes or operons. In agreement, reduction of the iron cofactor-dependent nitrate respiratory metabolism was detected. An adaptation of the central metabolism to lactate, citrate and amino acid as carbon sources with the induction of the glyoxylate bypass was observed, while other components of AUM like urea and creatinine were not used. Amino acid utilization pathways were found induced, while fatty acid biosynthesis was reduced. The high amounts of phosphate found in AUM explain the reduction of phosphate assimilation systems. Increased quorum sensing activity with the parallel reduction of chemotaxis and flagellum assembly underscored the importance of the biofilm life style. However, reduced formation of the extracellular polysaccharide alginate, typical for *P. aeruginosa* biofilms in lungs, indicated a different biofilm type for urinary tract infections. Furthermore, the obtained quorum sensing response results in an increased production of virulence factors like the extracellular lipase LipA and protease LasB and AprA explaining the harmful cause of these infections.

## Introduction

Urinary tract infections (UTIs) are one of the most common community-acquired bacterial infections. Moreover, in association with indwelling urethral catheters they are also the most common hospital-acquired infection responsible for 40% of all nosocomial infections [1], [2]. In 82% of the cases, uncomplicated UTIs are caused by *Enterobacteriaceae*. However, in complicated UTIs the Gram-negative proteobacterium *Pseudomonas aeruginosa* plays a major role [3], [4], [5]. It was identified as the dominant agent in 35% of catheter-associated UTIs (CAUTIs) and as the third most frequent pathogen of complicated UTIs [6], [7].

Strains of *P. aeruginosa* are known for their enormous versatile and adaptive physiology. In general *P. aeruginosa* can be regarded as a successful environmental bacterial genus with the human body as one of its habitats. Because of its high intrinsic antibiotic resistance and its ability to develop new resistances during antibiotic treatment, infections with *P. aeruginosa* are difficult to eradicate [8]. The progressive course of these infections is due to the multifactorial virulence of the bacterium [9], [10]. *P. aeruginosa* produces a variety of extracellular enzymes, which alone or synergistically with others, are causing cell death and necrosis in the human host [9], [11], [12]. Strains deficient in some of these factors were found to be less virulent [13], [14]. One major known strategy for the successful infection of the urinary tract is the formation of biofilms at the urethral epithelium or on the surface of urethral catheters [15]. Stable surface attached biofilms provide protection against harmful environmental conditions [16]. In the urinary tract these include mechanical shear stress, host immune response, limitation of iron, nutrients and oxygen, and antibiotic treatment. The biofilm life style offers the basis for community-associated job sharing functions during nutrient acquisition, protection, persistence and proliferation. Alginate as the major extracellular polysaccharide mediates the stability of *P. aeruginosa* biofilms in the cystic fibrosis lung [17], [18]. Mucoid strains overproducing alginate were found dominant in lung infections, however, in urinary tract infections only 1% of the *P. aeruginosa* isolates showed mucoid phenotype [19], [20]. In non-mucoid strains additional polysaccharides synthesized by enzymes encoded by the *pel-* or the *psl*-operon accomplish the structural stability of biofilms [21].

For infections of the cystic fibrosis lung it was demonstrated that virulence factor synthesis and biofilm formation are regulated by complex quorum sensing (QS) systems. Currently, three major QS systems are known for *P. aeruginosa* [22], [23], [24]. The hierarchically organized LasRI and RhlRI systems utilize various acyl-homoserine lactones as signal molecules [22]. The third system utilizes 2-heptyl-3-hydroxyl-4-quinolone [22], [23]. This *Pseudomonas* quinolone signal (PQS) is involved in infection response [24], [25]. It acts as a coinducer for the LysR-type PQS regulator PqsR also called MvfR, which positively regulates the transcription of several virulence factors (e.g. *pvd* and *pch* expression for the biosynthesis of siderophores) and the PQS biosynthesis operon *pqsABCDE* itself [24], [26], [27]. Previous investigations showed that the production and utilization of virulence factors in *P. aeruginosa* is infection site-specific [10], [28], [29].

It was reported that iron-limitation has a significant influence on the virulence of uropathogenic *P. aeruginosa* strains [30]. A complex regulatory network is necessary to control iron homeostasis at the level of transcription. In *P. aeruginosa* the transcriptional ferric uptake regulator (Fur) negatively controls the expression of genes involved in the acquisition of environmental iron including the receptors, binding proteins and transporters for the Fe(III)-chelating siderophores pyochelin and pyoverdine, as well as the heme binding hemophores. The Fur regulatory cascade also involves several extracytoplasmatic function (ECF) sigma factors (reviewed in [31], [32]). One important ECF sigma factors is PvdS, which directly activates the expression of the biosynthesis genes of pyoverdine under iron-limited conditions [33], [34].

Finally, oxygen-limitation plays an important role in infection-relevant habitats and also for the process of UTIs [35], [36]. The urinary tract is a highly heterogeneous environment concerning oxygen tension and consists of microaerobic to anaerobic niches [37]. Moreover, it is well-known that an oxygen tension gradient exists even in aerobically grown biofilms. Already below the surface layers of a biofilm, anaerobic conditions arise through the respiration of metabolic active microorganisms, increasing their antibiotic tolerance [38]. *P. aeruginosa* is a facultative anaerobic bacterium with enormous metabolic capabilities. Under aerobic conditions it utilizes various sugar derived carbon sources via the Entner-Doudoroff-Pathway and performs highly developed oxygen respiration using multiple terminal oxidases [39], [40]. In the absence of oxygen and the presence of nitrate or nitrite *P. aeruginosa* is able to grow by denitrification [41], [42]. Moreover, it uses arginine fermentation to produce energy under anaerobic conditions in the absence of alternative electron acceptors. Finally, during anaerobic energy limitation a mixed acid type fermentation is employed, which does not allow growth but sustains long-term anaerobic survival and is essential for microcolony formation of *P. aeruginosa* [43], [44], [45].

The urinary tract is a unique environment characterized by low iron content, carbon sources like citrate, lactate and amino acids and the presence of uric acid and urea [46], [47]. It is known that *P. aeruginosa* prefers short-chain fatty acids, amino acids and polyamines as C-sources [48], [49]. The utilization of urine as nutrient source was not investigated so far. New metabolic and corresponding regulatory strategies for urinary tract biofilms can be expected.

The urine composition can significantly vary between different humans based on sex, age, diet, or time of day making reproducible systematic analyses of urinary tract infections difficult [46], [47]. Artificial urine medium (AUM) provides the basis for the reproducible systematic investigation of urinary tract specific molecular adaptation strategies of pathogens. Consequently, we used AUM to elucidate the regulatory and metabolic network specific to the adaptation of *P. aeruginosa* biofilms to the urinary tract.

## Results and Discussion

### Anaerobic Biofilm Growth of *P. aeruginosa* Under Artificial Urinary Tract Conditions

The aim of this study was the investigation of the adaptive molecular strategies and the underlying regulatory network of *P. aeruginosa* grown under urinary tract-specific conditions examined by comprehensive transcriptome, proteome and metabolome analyses. Therefore, a standardized, reproducible *in vitro* growth system mimicking urinary tract infection-relevant conditions was developed using an artificial urine medium (AUM) simulating the averaged urine of an human healthy adult [46]. In general, this is a phosphate-buffered urea solution containing low amounts of lactate, citrate and uric acid, amino acids and high amounts of ammonium chloride and sodium sulphate [46]. To identify the urinary tract specific behavior of *P. aeruginosa* cells a comparison of the obtained data to data obtained for standardized normal growth environment was necessary. In our system, the biofilm growth rate of *P. aeruginosa* PAO1 under anaerobic conditions on AUM was highly similar to the growth on 10-fold diluted LB medium (Table 1 and Figure S1). The viable cell counts at the beginning of the stationary phase were comparable in both media. Alternatively, several other standard media like M9 minimal media with various C-sources were tested. However, all tested media failed to sustain growth as observed for AUM (data not shown). To exclude growth rate related phenomena we selected 10-fold diluted LB as a reference medium. For the experiments outlined below, late logarithmic phase biofilms were used in analogy to the phase growth observed within the urinary tract by transcriptome analyses performed for *E. coli* in a UTI mouse model [50].

**Table 1:** Growth of *P. aeruginosa* PAO1 at 37°C as biofilms on membrane filters placed on the surface of agar media containing AUM or 10-fold diluted LB, respectively.

mediuma)	OD_578_ stat. phase	growth time to stat. phase [h]	growth rate µ [h^−1^]	viable cells/ml b) c)
AUM	0.53	24	0.300	1.01×10^8^
1∶10 LB	0.68	22	0.402	1.19×10^8^

The results are expressed as mean value of three independent experiments performed in duplicates.

a) For anaerobic cultivation the media were supplemented with 50 mM nitrate.

b) The biofilm mass of one filter was resuspended in 1 ml 0.9% (w/v) NaCl solution.

c) Membrane filter circular surface: 4.91 cm^2.^

*In vivo*, urinary tract infections are caused by bacterial cells attached to the urethral epithelium or catheters in form of biofilms [51]. Within this habitat microaerobic and anaerobic zones were found [37], [38]. Consequently, *P. aeruginosa* was grown anaerobically on the surface of membrane filters placed on nutrient agar plates. The used cultivation system resulted in so called unsaturated biofilms, which are routinely used in biofilm research [52], [53], [54]. Replacing the membrane filter biofilms every day on fresh nutrient agar plates simulated the regular urine flow. Similar growth systems mimicking the *in vivo* conditions using artificial media were successfully used to investigate the process of lung infections caused by *P. aeruginosa* [55], [56].

### Systems Biology Methodology for Transcriptome, Proteome and Metabolome Determination

We intended to systematically determine the flux of cellular information typical for urinary tract adapted *P. aeruginosa* biofilms. Transcriptome analyses using *Pseudomonas* GeneChips® from Affymetrix were performed. The statistical analysis of the data indicated a high quality of hybridisation with a low background and a low variation between the three biological replicates. Moreover, most of the genes organized in operonic structures were expressed in a similar manner. To focus on the major adaptation processes, we initially analysed genes differently expressed with a fold change cut-off of 2.0 in pairwise comparison (Table S1). In some cases, genes differently expressed up to 1.5-fold were considered additionally. In total, 1019 genes were found differently expressed (2.0-fold) in AUM grown biofilms indicating a complex adaptation process of *P. aeruginosa* biofilms to the urinary tract (Table 2).

**Table 2:** Numbers of genes, proteins and metabolites of *P. aeruginosa* PAO1 differently expressed in biofilms grown on AUM compared to 10-fold diluted LB grown biofilms.

	Increased	Reduced	Total
Transcripts	161	149	310
Proteins	6	6	12
Metabolites	27	7	34

Furthermore, proteome analyses via 2D-gelelectrophoresis with subsequently MALDI-TOF analyses (Table S5) and metabolome analyses by gas chromatography mass spectrometry (GC-MS) were performed (Table S6). Our results revealed approximately 400 detectable proteins in the pH range between 3 and 10 derived from *P. aeruginosa* biofilms. However, only 19 proteins were found differently produced in biofilms grown on AUM in comparison to those grown on 10-fold diluted LB (Table 2). The high cut-off of 10.0 employed for the proteome analyses to obtain robust results, identified proteins almost exclusively present in one of the analyzed condition. This explains the relatively low number of considered proteins. Similarly, a robust approach was taken for the metabolome analyses. In total, approximately 150 metabolites were detected by GC-MS analysis of *P. aeruginosa* biofilms and matched to known compounds by comparison with metabolite libraries [49]. Out of these, 34 metabolites were found differently produced in AUM compared to 10-fold LB grown biofilms (Table 2 and Table S6). In general, the protein amount and the mRNA copy numbers for any gene in a single cell is uncorrelated and moreover, underlay different turnover periods between few seconds and several hours [57]. However, our data of the different approaches fit very well to each other. More than 70% of the proteins and also 60% of the metabolome data matched to the transcriptional response of the cells. However, since only 5 metabolic active enzymes were identified by proteome analysis only 26% overlap was found to the metabolome data. Nevertheless, obtained results from tanscriptome, proteome and metabolome analyses were sorted and interpreted using various outlined bioinformatic approaches to unravel the regulatory and metabolic networks involved in the adaptation of *P. aeruginosa* to the urinary tract.

### Iron Limitation Causes the Major Adaptation in Biofilms under Urinary Tract Conditions

**Figure 1:**
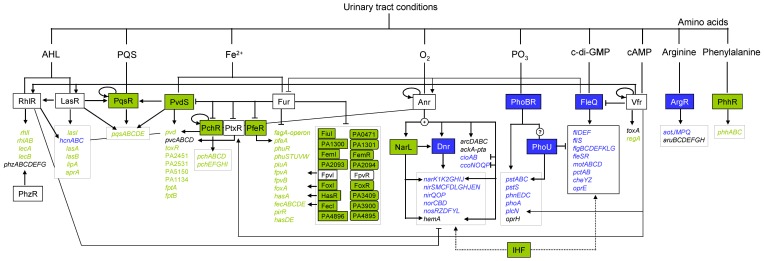
Regulatory network for the adaptation of *P. aeruginosa* to urinary tract conditions. Pairwise comparisons of the gene regulatory networks of biofilms anaerobically grown on AUM and 10-fold diluted LB were performed. For this purpose competitive transcriptome data were bioinformatically integrated into the shown regulatory network. The major regulatory signals present in the urinary tract detected by a set of global regulators controlling urinary tract related gene expression are summerized. AHL, homoserine lactone; cAMP, cyclic adenosine monophosphate; c-di-GMP, cyclic-di-guanosine monophosphate; PQS, Pseudomonas Quinolone Signal. Arrows: positive regulation, T-line: negative regulation; green: induced, blue: reduced, white: not differently expressed.

The majority of genes differentially expressed in biofilms grown under anaerobic AUM conditions were related to acquisition of iron (Table S2). The 86 induced genes encoded 25 regulators, 23 transporters, 29 biosynthetic enzymes and 9 proteins of other or unknown function. The overall observed gene regulation principle indicated strong iron limitation under AUM growth conditions. Using the PRODORIC database [58] for interpretation of the data and ProdoNet [59] for visualisation, a complex regulatory network active under urinary tract-like conditions was deduced (Figure 1). On the top of the regulative cascade the Fe(II)-sensing Ferric uptake repressor Fur was identified. In the presence of Fe(II) Fur directly represses multiple genes involved in iron uptake. According to the iron limitation in the AUM, Fur-repressed genes were found de-repressed. For example, the operon PA4468–PA4471 was found induced in AUM up to 22-fold. This operon consists of the Fur-associated gene *fagA*, the superoxide dismutase gene *sodM* and the iron sulfur cluster free fumarase gene *fumC1* [60]. SodM and FumC1 proteins were also found exclusively present in AUM grown biofilms via proteome analyses. In agreement, the promotor of the *fag*-operon contains two Fur-boxes for Fur binding [60]. Further Fur-regulated operons (PA4708–PA4710 and PA3407–PA3408) encoding for the heme transporter PhuT, the cytoplasmic heme binding protein PhuS, the heme/hemoglobin uptake outer membrane receptor PhuR, the hemophore HasA and the outer membrane hemophore receptor HasR were found up to 64-fold induced in AUM grown biofilms (Table S2). Increased PhuR protein production in AUM grown biofilms was also detected via proteome analyses (Table S5). Fur-regulation of the heme uptake operons *phu* and *has* were reported before in detail [61], [62]. Several bacterial pathogens, including *P. aeruginosa* can obtain iron by the degradation of heme or heme-containing compounds [63]. It was postulated that HasA is indirectly regulated via QS through the processing of the HasA-preprotein by the QS-regulated extracellular proteases AprA, LasA and LasB [64], [65]. Interestingly, the *lasB* (PA3724) and the *aprA* (PA1249) genes also were found induced in AUM grown *P. aeruginosa* biofilms. This indicates a complex regulatory circuitry to acquire iron by the transport and degradation of heme. The nature and role of QS is described below.

**Figure 2:**
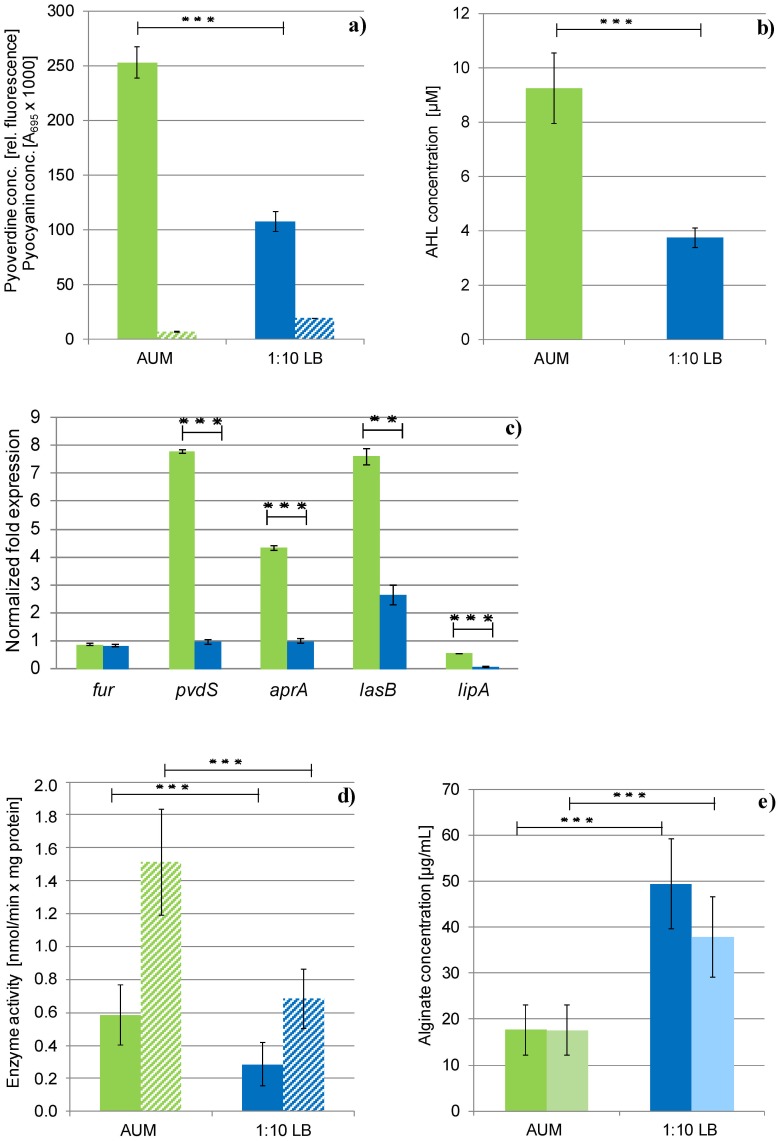
Virulence factor production under urinary tract conditions. a) Determination of pigments. The siderophore pyoverdine (uni) and the redox-active pyocyanin (stripes) were measured in supernatants of anaerobically grown *P. aeruginosa* PAO1 from AUM (green) and 10-fold diluted LB cultures (blue). The results are expressed as mean value +/− standard deviation (SD) of three independent experiments performed in triplicates. *** *p* = 0.04. b) Quorum sensing activity of *P. aeruginosa* grown anaerobically in biofilms on AUM (green) or 10-fold diluted LB (blue). The results are expressed as mean value +/− SD of three independent experiments performed in triplicates. *** *p* = 0.002. c) Expression of the *fur*, *pvdS*, *aprA*, *lasB* and *lipA* genes quantified via qRT-PCR. *P. aeruginosa* PAO1 was grown as colony biofilms under anaerobic conditions on AUM (green) or 10-fold diluted LB (blue) at 37°C up to the late logarithmic growth phase. Both media were supplemented with 50 mM nitrate to sustain anaerobic denitrifying growth. The results are expressed as mean value +/− SD of three independent experiments performed in triplicates. d) Activities of extracellular protease (dark) and lipase (light) detected in supernatants of anaerobically grown *P. aeruginosa* PAO1 biofilms on AUM (green) and 10-fold diluted LB (blue). *** *p*<0.05; ** *p*<0.5. e) Concentration of the exopolysaccharide alginate produced in biofilms of *P. aeruginosa* grown under anaerobic conditions on AUM (green) or 10-fold diluted LB (blue). The results are expressed as mean value +/− SD of three independent experiments performed in duplicates. *** *p*<0.005.

Furthermore, Fur represses 10 extracytoplasmatic function (ECF) alternative sigma70-factors and their corresponding transmembrane sensor encoding genes [32], [66], [67]. These were found induced up to 12.0-fold in AUM biofilms (Table S2). Several of the known ECF sigma-factor target genes were co-induced (Figure 1) confirming the proposed regulatory cascade [68], [69]. One of the ECF-sigma factor genes regulated by Fur is *pvdS,* which was found 6.6-fold induced. PvdS in turn controls the transport of iron into the cell via transcriptional regulation of genes involved in iron uptake including the biosynthesis *pvd*-genes of the siderophore pyoverdine [70], [71]. Consequently, the *pvd*-gene cluster was strongly induced in AUM grown biofilms (Table S2). Pyoverdine binds Fe(III) and is transported by the specific receptor FpvA through the outer membrane [72], [73]. Accordingly, significant amounts of the iron-chelating siderophore pyoverdine were detected by biochemical experiments for cells grown on AUM in relation to biofilms grown on 10-fold diluted LB (Figure 2a).

*P. aeruginosa* produces a second Fe(III)-chelating siderophore in response to iron-starvation called pyochelin. Pyochelin-bound iron is transported into the periplasm of *P. aeruginosa* via the specific outer membrane receptor FptA [74], [75]. FptA protein was detected exclusively in AUM grown biofilms (Table S5). Furthermore, the whole pyochelin biosynthesis *pch*-gene cluster as well as the *fptA* gene was found up regulated in AUM grown biofilms. The whole cascade is controlled indirectly by Fur via repression of *pchR* regulator gene [76]. Again, major parts of this regulatory network were found induced (Figure 1). To verify the results of the DNA microarray analysis, quantitative RT-PCR (qRT-PCR) was performed for the main regulators Fur and PvdS (Figure 2c). As expected, higher amounts of transcripts of the *pvdS* gene were observed under urinary tract conditions, while the *fur* gene was not differentially expressed. However, this regulator is known to be expressed constitutively, to allow a fast response to changes in iron availability [70].

Interestingly, comparable results were obtained during DNA microarray analyses of iron starved *P. aeruginosa* shake flask cultures [66], [67]. The overlap with our experiments is approximately 80% with respect to the induced genes. Differences between the two iron-related transcriptional profiles [66], [67] and the AUM-induced iron starvation response lie mostly in genes, which do not possess a Fur-binding box, as for example the *cyo*-operon (PA1317–PA1321), *gabT* (PA0266), or PA4633 encoding a potential chemotaxis transducer. These genes were found up regulated in the experiment performed by Ochsner and coworker but not in our experiments [66]. Moreover, they found the genes coding for a probable ferrous iron transporter *feoAB* (PA4357–PA4359) highly induced [66]. Since it was reported that these genes were Mg^2+^-regulated via the two-component system PmrAB [77], we did not found them induced in our experiment. However, many Fur-regulated genes were also found induced under oxidative stress in hydrogen peroxide treated cultures, which was explained by the loss of iron from the Fur-Fe^2+^ complex by the action of reactive oxygen species [78]. In agreement, we detected a significant overlap between the AUM and the hydrogen peroxide induced transcriptional profiles. Our results mirror quite well the conditions in the urinary tract, with respect to iron limitation [30], [79]. Also *E. coli* grown in the urinary tract of mice revealed significantly enhanced expression of genes of iron acquisition [50]. Finally, also during lung infections *P. aeruginosa* suffers from iron limitation indicated by highly induced iron acquisition systems [56], [80], [81]. However, only few studies were done so far showing rather the production of pyochelin instead of pyoverdine or heme acquisition systems [56], [80], [81].

In summary, the results suggest that *P. aeruginosa* senses a strong iron limitation under urinary tract-like conditions and adapts to this stress via a complex and fine-tuned gene regulatory network. This observation provides evidence that the artificial *in vitro* growth system used here nicely reflected the *in vivo* conditions in the urinary tract.

### The Respiratory Energy Metabolism of Biofilms is Reduced under Anaerobic Urinary Tract Conditions by Iron Limitation and Quorum Sensing

For anaerobic growth *P. aeruginosa* usually generates energy via denitrification using nitrate instead of oxygen as terminal electron acceptor during respiration [41]. Consequently, operons (*nar, nir, nor, nos, hem, moa*) encoding the corresponding enzymatic machineries were found induced in anaerobically grown biofilms in diluted LB medium. Surprisingly, the transcription of genes encoding proteins involved in denitrification, as the nitrate transporter *narK*, the nitrate reductase encoding *nar*-operon, the nitrite reductase encoding *nir*-operon, the NO reductase encoding *nor*-operon, the nitrous dioxide reductase encoding *nos*-operon and the regulator *nosR* was found significantly reduced in anaerobically grown AUM biofilms (Table S2 and Figure 1). In combination with a decreased expression of the genes encoding the microaerobically active cbb_3_-type cytochromes *cooM*, *ccoN*, *cooO*, *cooP* and *cooQ* (PA1553–PA1557) a reduced anaerobic respiration under urinary tract conditions can be proposed.

**Figure 3:**
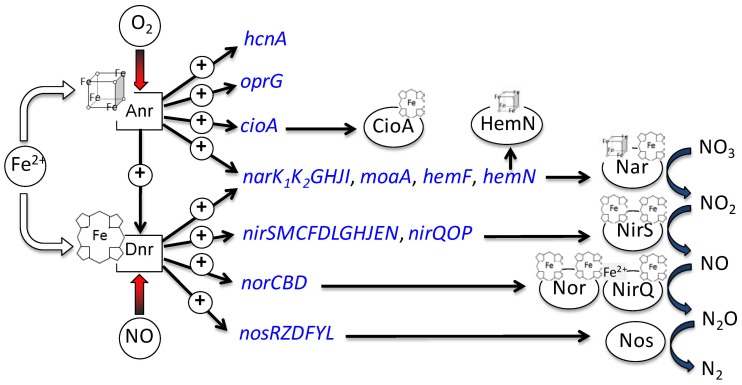
Influence of iron availability on the formation of the anaerobic respiration apparatus. Pairwise comparisons of transcriptome data between biofilms grown anaerobically on AUM and 10-fold diluted LB supplemented with 50 mM nitrate were performed. Expression of genes involved in anaerobic respiration found reduced in anaerobically grown biofilms on AUM are shown. Iron is essential for cofactor synthesis required for the anaerobic regulators Anr and Dnr as well as various enzymes of denitrification.

**Figure 4:**
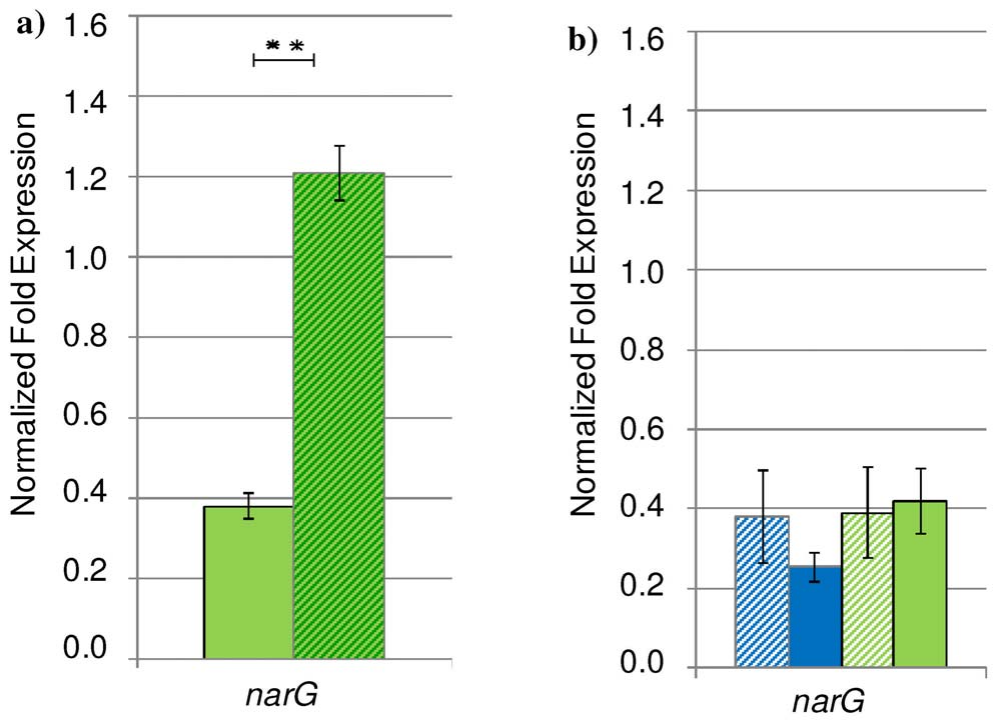
Expression of the nitrate redutase gene *narG* quantified via qRT-PCR. *P. aeruginosa* PAO1 was grown as colony biofilms under a) anaerobic conditions on AUM supplemented with 50 mM nitrate in the absence (uni) and presence of additional 7 mM iron (strips) and b) aerobic conditions on AUM (green) or 10-fold diluted LB (blue) in the absence (strips) and presence (uni) of 25 mM nitrate without additional iron at 37°C up to the late logarithmic growth phase. The results are expressed as mean value +/− SD of three independent experiments performed in triplicate.

Several of the enzymes involved in the denitrification process contain multiple ^hello^4Fe-4S]^2+^-clusters, heme or siroheme as cofactors and are therefore, strictly iron-dependent in their function [41]. Moreover, the global oxygen-sensing regulator Anr contains a ^hello^4Fe-4S]^2+^-cluster. The Dnr (dissimilative nitrate respiration) regulator directly involved in transcriptional regulation of denitrifying enzyme-encoding genes contains heme for NO-sensing [82]. Therefore, we concluded that the induction of the genes for the denitrification pathway failed under iron-limited conditions of the urinary tract due to significantly reduced activity of the cofactor containing Anr and Dnr (Figure 3). qRT-PCR analyses were additionally performed for *narG* expressed in biofilms grown under anaerobic conditions in AUM supplemented with additional 7 mM iron (Figure 4a). Significant enhanced amounts of *narG* transcript in the iron-enriched culture conditions were detected, which evidenced the close connection between the anaerobic respiration and iron acquisition. As expected, under aerobic conditions the expression of *narG* was found comparable in AUM and 1∶10 diluted LB in the presence and absence of nitrate Figure 4b). Moreover, the amount of the *narG* transcript was comparable to the amount detected under anaerobic conditions in AUM without additional iron. This confirmed the DNA-microarray results indicating a minor role of denitrification in anaerobically AUM grown biofilms.

Previous growth phase-depended DNA-microarray analysis of QS-mutants showed the negative influence of QS on the expression of the *nos*-, *nor*-, *nar*-, and *moe*-operons of *P. aeruginosa* [83]. Corresponding regulator RhlR binding sites were identified in the promoter regions of these operons [83]. Accordingly, the low expression of these operons under anaerobic conditions in AUM biofilms can additionally be explained by these findings since QS were found induced in AUM grown biofilms (Figure 1, see below). In contrast, during lung infections as well as under artificial lung conditions an enhanced anaerobic respiration and reduced QS activity were observed [35], [56].

The questions for the energy metabolism sustaining anaerobic growth of AUM biofilms remain mysterious. Interestingly, ORF PA0751 similar to the *amoA* gene of *P. putida*, encoding ammonia oxidase was found strongly induced (9.0-fold) in anaerobic AUM biofilms. However, currently there is no experimental evidence for ammonia oxidation in *P. aeruginosa*.

### Iron Limitation-dependent Quorum Sensing is Induced in AUM Grown Biofilms

It is well established that iron limitation is a major signal of the quorum sensing (QS) system [84]. In agreement, QS-active AHL-reporter strains showed significantly higher AHL concentrations in anaerobic AUM cultures in relation to 10-fold diluted LB grown cultures (Figure 2b). Moreover, in our metabolome analyses decreased concentration of 5-methylthioadenosine (5-MTA) was detected in AUM grown biofilms (Table S6). 5-MTA is a typical intermediate of the methionine metabolism, which is required for the synthesis of QS-active AHLs [85]. The decreased concentration of this intermediate gave a hint on the enhanced flow of this pathway. Although the two genes coding for the two QS-system regulators RhlR and LasR were not found differently expressed, the genes for the two AHL synthases LasI and RhlI were slightly induced in AUM grown biofilms (Table S2 and Figure 1). Furthermore, QS-controlled genes *lipA*, *lasB* and *aprA* encoding extracellular enzymes of *P. aeruginosa* were found induced in AUM-grwon biofilms (see below). Moreover, in dependence of iron limitation the sigma factor PvdS positively regulates the multiple virulence factor regulator MvfR (also named PqsR). PqsR and PvdS cooperatively activate the expression of the PQS biosynthesis gene cluster *pqsABCDE*. Usually the transcription of *pqsR* is also triggered by the QS regulator LasR [86]. In agreement, the PQS mediating genes *pqsABCDE* (PA0996–PA1000) and *pqsR* (PA1003) were found induced in anaerobically grown AUM biofilms (Table S2).

Altogether, an enhanced cell-cell communication via QS for biofilms grown under urinary tract infection-relevant conditions can be proposed. Interestingly, in acute lung infections caused by *P. aeruginosa* QS-active AHL concentrations can also be detected. However, in chronic lung infections QS was found reduced [56], [87]. Moreover, the complete loss of QS by an enhanced mutation of the *lasR* gene was reported for late stage CF lung isolates [80], [88].

### Urinary Tract Conditions Lead to Increased Virulence Factor Production

A close relationship between the control of virulence genes, iron-limitation and corresponding quorum sensing was reported before [30], [70], [84]. The two QS systems of *P. aeruginosa* (RhlRI and LasRI) control the gene expression of several extracellular enzymes by an AHL-dependent binding of the regulators to the promoter regions of the corresponding target genes [22], [89]. In agreement with the detected enhanced QS activity, the expression of several virulence factor genes was found induced in anaerobically AUM grown biofilms (Table S2). Enhanced expression of the genes encoding for the type II secreted lipase LipA and its chaperone LipH (2.1-fold), the esterase EstA, the lectin LecA, the chitinase ChiC, the proteolytic elastase LasB, the alkaline protease AprA and the protease PasP were detected. The enhanced gene expression of *aprA*, *lasB* and *lipA* was verified by qPCR (Figure 2c). These genes were reported to be QS-regulated before [90].

In accordance with the gene expression data, increased activities of lipases and proteases were measured for AUM grown cultures by biochemical assays (Figure 2d). These exoenzymes usually degrade macromolecules extracellular for nutrition of the bacterium. Obviously, an enhanced extracellular protein and lipid degradation is essential for bacterial growth under the nutrient-limited conditions in the urinary tract. *In vivo*, these activities led to tissue damages [9]. Consequently, enhanced production of such extracellular enzymes might explain the progressive course of *P. aeruginosa* urinary tract infections. Interestingly, a decreased production of virulence factors was observed in chronic lung infections caused by *P. aeruginosa* [91]. In contrast to urinary tract infections, the lung was described as nutrient rich environment [92].

### Alginate is not a Major Constituent of AUM Biofilms

QS is also indirectly involved in the regulation of alginate production [93], [94]. Usually, alginate mediates the structure and the stability of *P. aeruginosa* biofilms and could be also important for the formation of biofilms under urinary tract conditions [95], [96]. Surprisingly, low alginate concentrations were observed for AUM grown biofilms (Figure 2e) indicating that the stability of biofilms in the urinary tract is not dependent on the production of this polysaccharide. The alginate regulator protein AlgR in combination with the alternative sigma factor AlgU usually activates the expression of alginate biosynthesis [97]. The alginate biosynthesis genes are further repressed by the anti-sigma factor MucA. MucA inactivates AlgU at the posttranslational level [98]. In agreement with our findings of reduced alginate formation under AUM conditions, the AlgR protein was exclusively detected in 10-fold diluted LB grown biofilms, while the MucA protein was solely observed in AUM grown biofilms (Table S5). These data might explain the gene regulatory scenario underlying the observed reduced alginate production. The expression of the *pel-* and *psl-*operons mediating the biosynthesis of two other exopolysaccharides in *P. aeruginosa* remained unaffected under tested conditions. Clearly, these results indicate that the stability of infection-relevant biofilms under urinary tract conditions is mediated by other factors. Recently, a *P. aeruginosa* exopolysaccharide biosynthesis mutant study revealed the systematic replacement of alginate by extracellular DNA during biofilm formation [99]. In agreement with these observations, extracellular DNA was microscopically observed in AUM grown biofilms. Destruction of these biofilms by DNase treatment was demonstrated/data not shown). Obviously, the urinary tract infection scenario is in contrast to *P. aeruginosa* caused lung infections, where alginate production was found increased [80], [100]. As a consequence, most chronic isolates from infected lungs convert to the mucoid phenotype, whereas only 1% of the isolates from UTIs were found alginate-overproducing [101].

### Adaptation of the Central Metabolism

The adaptation of the central metabolism is closely related to the C-sources supplied by the AUM medium. Interestingly, urea and creatinine, the main components of urine, were no growth substrates when tested as sole carbon source (Table 3). In agreement, neither the gene encoding for the urease of *P. aeruginosa* nor the genes for the enzymes of the urea cycle were found induced in AUM grown biofilms (Table S1). However, urea was not detected by GC-MS analyses in cells grown in urea containing AUM (Table S6). Genes for enzymes involved in creatinine metabolism are missing in the genome of the tested *P. aeruginosa* strain.

**Table 3:** Growth of *P. aeruginosa* in liquid cultures at 37°C and 200 rpm under aerobic conditions in 10-fold diluted LB, AUM and in AUM prepared with only one single C-source each.

	Growth rate µ [h^−1^]
1∶10 LB	1.20±0.22
AUM	1.21±0.24
Lactate	0.39±0.04
Citrate	0.56±0.11
Uric acid	0.29±0.14
Peptone	0.96±0.18
Urea	0.0±0.0
Creatinine	0.0±0.0

The results are expressed as mean value +/− SD of four independent experiments performed in duplicates.

Citrate, lactate and various amino acids derived from peptone were obviously utilized. Growth experiments using AUM supplemented with these compounds as sole C-sources showed that *P. aeruginosa* was able to utilize amino acids (from peptone) and other organic acids like citrate and lactate (Table 3). These compounds are channelled into the central metabolism close to or even directly at the level of the citric (TCA) cycle. Accordingly, several metabolites of the TCA cycle like α-ketoglutarate, fumarate, malate and intermediates of the gluconeogenesis pathway as 3-phosphoglycerate and phosphoenolpyruvate were detected in higher amounts in AUM grown biofilms in (Table S6). Furthermore, the cis-aconitate porin OpdH was detected in AUM grown biofilms by proteome analyses (Table S5). Genes involved in the TCA cycle as *gltA/prpC* encoding citrate synthase 1 and 2, *icd* encoding an isocitrate dehydrogenase were found induced (Table S3). Also, the expression of the *aceB* gene for the malate synthase of the glyoxylate shunt was enhanced. Interestingly, transcription of genes of glycolate degradation (*glcB*, *glcDEFG*, PA1499–PA1502) was also found induced. This pathway converts glycolate via glyoxylate to C_3_-compounds compatible with the gluconeogenesis pathway. Obviously, *P. aeruginosa* utilized this pathway for the transformation of glyoxylate into glycerate since AUM did not contain glycolate.

Consequently, gluconeogenesis is required for pentose formation to allow for nucleic acid biosynthesis. Intermediates of the gluconeogenesis pathway as 3-phosphoglycerate and phosphoenolpyruvate were detected in higher amounts in anaerobically AUM grown biofilms (Table S6). Furthermore, we observed a drastic reduction of the nitrate respiratory chain formation due to iron limitation which might lead to a decrease in NADH+H^+^ oxidation (see above). Therefore, utilization of the glyoxlate shunt provides the necessary precursors for gluconeogenesis and partially prevents further NADH+H^+^ formation via the citric cycle. Similarly, some enzymes of the TCA cycle possess iron-containing cofactors. Iron-dependent is the aconitases, the succinate dehydrogenase and the fumerase A. All of the corresponding genes (*acnA*, *acnB*, *sdhCDAB*, *fumA*) were found down regulated under iron-limited conditions in AUM (Table S3). Several of these genes are controlled by iron via the Fur-regulated small RNAs of the PrrF type [31]. Fumerase A is then replaced by iron-free fumerase C1 (*fumC1*). The *fumC1* gene was found highly up regulated (18-fold) in AUM grown biofilms. Furthermore, also the FumC1 protein was detected exclusively in AUM grown biofilms (Table S5). The *fumC1* containing operon is regulated by the iron-sensing repressor Fur (see above; [70]). In summary, complex gene expression and metabolite profiles were observed for the TCA cycle and glyoxylate shunt, most likely reflecting various overlapping adaptation processes.

### Enhanced Amino Acid Metabolism under Urinary Tract-like Conditions

It is well known, that *P. aeruginosa* has a preference for amino acids as carbon and nitrogen source [48], [49]. The amount of peptides is quite similar for both media used (1.25 g/l in AUM and 1.5 g/l in 10-fold diluted LB). However, significantly higher concentrations of amino acids including alanine, ß-alanine, aspartarte, glutamate, glutamine, serine and ornithine were detected in the intracellular metabolome of AUM grown biofilms (Table S6). The strong increase of the glutamine concentration in the cell is related to the nitrogen contents of the AUM medium influencing the glutamine/glutamate equilibrium of the cell. Moreover, together with the enhanced expression of the extracellular proteases *pasP*, *lasB* and *aprA* and several genes encoding for amino acid transporters like the sodium/proline symporter *putP*, the branched chain amino acid transporter *braC* and the pyroglutamate porin *opdO* and the exclusively production of the tyrosine porin OpdT an increased peptide and correspondingly amino acid turnover by AUM grown biofilms was deduced. This was supported by the finding of induced genes involved in amino acid metabolism (Table S4) and additional intermediates of the amino acid metabolism as 4-aminobutanoate, 5-aminopentanoate and others (Table S6).

**Figure 5:**
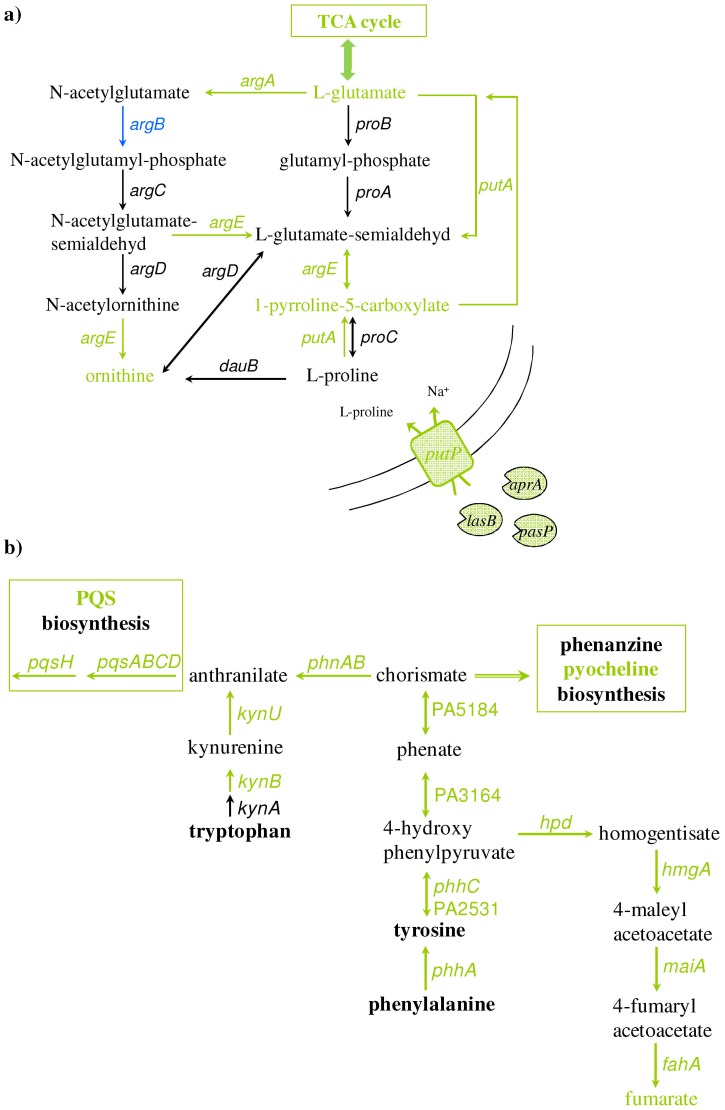
Degradation of aromatic amino acids as indicated by transcriptome and metabolome data. Degradation of a) proline and b) tryptophan, tyrosine and phenylalanine was shown. Pairwise comparisons of the transcriptome and metabolome data between biofilms grown anaerobically on AUM and 10-fold diluted LB supplemented with 50 mM nitrate were performed. Metabolite concentrations and gene expression levels found increased (green) or reduced (blue) in biofilms grown on AUM are displayed. Equally produced metabolites and expressed genes are marked in black.

The amount of intermediates of the proline degradation pathway as 5-oxoproline, and 1-pyrroline-3-hydroxy-5-carboxylate, glutamate and ornithine were found significantly elevated in anaerobic AUM biofilms (Figure 5a). The enhanced expression of genes involved in this process like the proline dehydrogenase gene *putA* (4.4-fold induction), the N-acetylglutamate synthase gene *argA* (1.7-fold induction), the acetylornithine deacetylase gene *argE* (1.6-fold induction) and the sodium/proline symporter gene *putP* (4.1-fold induction) in combination with observed metabolites indicated an enhanced proline uptake and degradation. The resulting intermediate glutamate can be directly channeled into the TCA cycle. Ornithine can be converted in several steps into arginine, which can be further utilized via the TCA cycle. In agreement, the glutaminase-asparaginase AnsB and its corresponding gene *ansB* were detected in anaerobically AUM grown biofilms. In *E. coli ansB* is regulated by the anaerobic regulator Fnr and cyclic-AMP [102] indicating that the cells in anaerobic AUM biofilms suffered from oxygen and energy-limitation as proposed before.

Furthermore, degradation pathways for the aromatic amino acids tryptophan, tyrosine and phenylalanine were found induced in AUM grown biofilms (Figure 5b). Throughout their degradation phenylalanine and tyrosine are converted into chorismate [103]. Chorismate can be subsequently converted into phenanzine or pyochelin or via anthranilate to PQS [103]. Accordingly, the *phhA*, *phhC* genes encoding a phenylalanine-4-hydroxylase and PA2531 encoding for an aromatic amino acid aminotransferases were found induced. Corresponding enzymes mediate the conversion of tyrosine and phenylalanine to 4-hydroxyphenylpyruvate (Figure 5b). The intermediate 4-hydroxyphenylpyruvate can subsequently be converted by the maleylacetoacetate isomerase MaiA, the fumarylacetoacetase FahA, the homogentisate 1,2-dioxygenase HmgA and the hydroxyphenylpyruvate dioxygenase Hdp to fumarate. The transcription of all of corresponding genes was found enhanced. Moreover, 4-hydroxyphenylpyruvate can be converted to chorismate in two sequential steps via the phenate dehydrogenase encoded by PA3164 and the chorismate dehydrogenase encoded by PA5184. Again, both genes were found to be induced. Subsequently, chorismate is converted by the anthranilate synthase PhnA to anthranilate, the direct precursor of the PQS [84]. Corresponding gene *phnA* was also found induced in AUM, A second way for anthranilate synthesis is the conversion of tryptophan [103]. The genes *kynU* and *knyB* encoding the enzymes involved in the degradation of this aromatic amino acid were found induced in AUM grown biofilms. In summary, these results suggest that besides the general utilization of amino acids as nutrients, an enhanced PQS activity of *P. aeruginosa* growing under urinary tract-like conditions is traceable. Accordingly, the genes involved in PQS biosynthesis were found enhanced in AUM (Table 3, see above).

Moreover, the transcription of genes involved in pyochelin biosynthesis (*pchDEFG*, *pchR*) was enhanced. Interestingly, pyocyanin biosynthesis genes were found reduced. In accordance, lower amounts of this blue pigment were measured in AUM cultures (Figure 1a). Pyocyanin is a blue redox-active secondary metabolite of *P. aeruginosa* with several cellular functions (reviewed in Lau et al., 2004). Moreover, it plays a role in pathogenesis by causing tissue damages by the production of reactive oxygen species and interferes with the immune system of the host [104], [105], [106]. However, these mechanisms seemed to play a minor role in urinary tract infections. In the light of this assumption, *P. aeruginosa* isolates from urinary tract infections were found frequently defective in pyocyanin production [10].

### Reduced Fatty Acid and Polyhydroxyalkanoate Biosynthesis

Most genes of fatty acid biosynthesis *(accA*, *accB*, *accC*, *fabAB*, *fabGD*) were found down regulated in anaerobic AUM grown biofilms (Table S1). In agreement, the C_16_ fatty acid derivative palmitic acid amide was found significantly decreased in its cellular concentration as documented by the metabolome analysis while the degradation products glycerol, phosphoethanolamine and O-phospho-serine were found significantly increased (Table S6). The derived formation of the storage metabolite polyhydroxyalkanoate (PHA) is blocked via down regulation of the *phaG* gene encoding 3-hydroxyldecanoyl-ACP-CoA transacetylase, the first enzyme of PHA biosynthesis. Overall, the energy consuming lipid formation is systematically reduced in anaerobic AUM grown biofilms.

### Urinary Tract Conditions Cause Reduced Phosphate Uptake and Utilization

Similarly, 14 genes involved in phosphate uptake were found down regulated in anaerobically grown AUM-biofilms. These include genes encoding for the phosphate-sensing two-component regulatory system PhoB/R, for phosphate transporters, the phosphate-specific porin OprO and for the alkaline phosphatase PhoA (Table S2). All these genes are usually expressed only under phosphate starvation [107], [108], [109]. Since human urine contains between 10 and 32 mM phosphate [47], AUM was buffered with the averaged phosphate concentration of 14 mM [46]. The results indicated that *P. aeruginosa* did not suffer from phosphate limitation under urinary tract conditions.

### Stress Response of Anaerobically AUM Grown *P. aeruginosa* Biofilms

Transcriptome analyses revealed the increased expression of typical osmotically induced genes like *osmC* (3.36-fold) and *osmE* (2.1-fold). In agreement, an enhanced osmotic stress response was observed for UTI causing *E. coli* in a mouse model [50]. Obviously, also AUM is inducing a mild osmotic response. Initially, we believed that the induced uptake (4.1-fold induction of the sodium/proline transporter gene *putP*) of the osmoprotective amino acid proline could be part of the osmo-response. However, metabolization of proline indicated by strongly induced proline degradation pathway (see above) relativized this assumption.

Furthermore, the superoxide-dismutase *sodM* was found 20-fold co-induced with genes of the Fur-controlled *fag*-operon (PA4471–PA4468). Anaerobic expression of superoxide stress genes was observed for *P. aeruginosa* and other bacteria before [110]. However, the second superoxide-dismutase encoded by *sodB* and the four catalases *katA*, *katB*, *katE* and *katN* were not differentially indicated as expected a minor relevance of oxidative stress under anaerobic conditions.

### Conclusion

**Figure 6:**
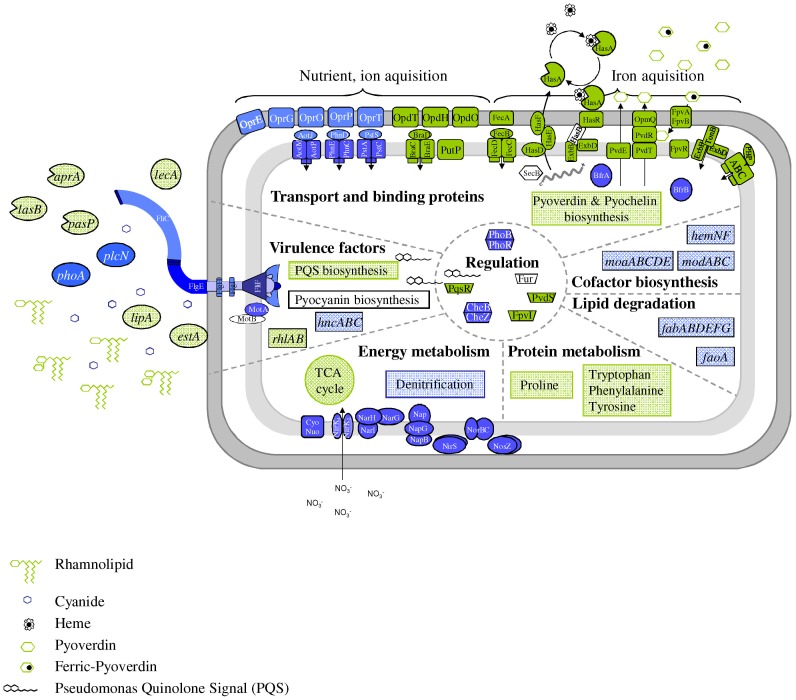
Regulatory and metabolic networks for the adaptation of *P. aeruginosa* to the anaerobic conditions in the urinary tract. The model presents the transcriptional, proteomic and metabolic response of *P. aeruginosa* grown under anaerobic conditions as colony biofilms on AUM. Transcriptome, proteome and metabolome analyses were performed as outlined in the text. Obtained data were integrated into the shown scheme. Expression of genes, protein concentration or pathways found induced (green), reduced (blue) or not differently expressed (white) in biofilms grown on AUM are shown.

To systematically investigate the adaptation of the opportunistic pathogen *P. aeruginosa* during urinary tract infections we investigated the transcriptome, proteome and metabolome of anaerobically grown biofilms on AUM. For summarizing the cellular adaptive processes active in AUM grown *P. aeruginosa* biofilms a schematic overview was given in figure 6. In contrast to the well-investigated lung infection by *P. aeruginosa*, infections of the urinary tract are dominated by the response of the bacterium to iron limitation. A large stimulon consisting of 86 genes including 25 regulatory proteins is induced. Moreover, the energy metabolism is limited by the lack of iron for essential cofactors. Biofilm formation is accompanied by the typical quorum sensing response including an enhanced expression of several extracellular enzyme-encoding genes. However, the extracellular polymers of biofilms are different for lung and urinary tract infections. Other typical urinary tract adaption strategies include the induction of various amino acid degradation pathways, reduced fatty acid biosynthesis and reduced phosphate uptake. Interestingly, major constituents of urine like urea and creatinine are not utilized by the bacterium. The determined unique molecular adaptation strategies of *P. aeruginosa* to urinary tract conditions indicate the need for the development of specifically adapted treatment strategies.

## Materials and Methods

### Bacterial Strains and Cultivation

The genome-sequenced model strain *P. aeruginosa* PAO1 [111] was used throughout this study. In previous studies we have demonstrated the surprising genetic and phenotypic heterogeneity of *P. aeruginosa* strains isolated from urinary tract infections [10]. As consequence we decided to utilize the best characterized *P. aeruginosa* strain PAO1. The bacterium was cultivated as colony biofilms on the surface of membrane filters placed on 1.5% (w/v) agar plates containing an artificial urine medium (AUM) described before [46] or 10-fold diluted Luria Broth (LB) as reference medium. For the growth of colony biofilms, membrane filters (cellulose acetate; diameter: 2.5 cm, pore size: 0.22 µm; Millipore, Eschborn, Germany) were inoculated with 50 µl of a LB-preculture with an OD_578_ of 0.002 and subsequently placed on the surface of the agar media. The biofilms were incubated for up to 6 days at 37°C under anaerobic conditions. The membrane filters were placed on fresh media every 24 hours. To enhance anaerobic cultivation the media were supplemented with 50 mM KNO_3_. Anaerobic liquid cultures used for pigment analysis were grown in 140 ml serum flasks filled to 95%. They were inoculated to an initial OD_578_ of 0.05. The growth of cultures was followed by optical density measurement at 578 nm and viable cell counts on LB agar determining colony forming units (cfu/ml).

### Preparation of RNA and cDNA Synthesis

Preparation of total RNA was performed as described before [112]. Total RNA was treated with 3 U RNase-free DNase I (USB Corporation, Cleveland, Ohio, United States) for 30 min at room temperature (RT) and further purified with RNAeasy mini kit (Qiagen, Hilden, Germany) according to manufacturer's instructions. RNA quality was determined with the Bioanalyzer 2100 (Agilent Technologies, Santa Clara, USA) and RNA 6000 Nano Kit (Agilent Technologies, Santa Clara, USA) according to manufacturer's instructions. Samples with RNA integrity numbers (RIN) higher than 8.5 were used. cDNA synthesis, fragmentation and labeling were performed according to protocols for the Affymetrix *Pseudomonas* GeneChip® with slight modifications as described before [43]. cDNA was purified with the QIAquick PCR Purification Kit (Qiagen, Hilden, Germany) according to manufacturer's instructions.

### Transcriptome Analysis

Affymetrix *Pseudomonas aeruginosa* GeneChips® (Santa Clara, California) were used. The Affymetrix Core Facility at the Helmholtz Centre for Infection Research (HZI), Braunschweig, Germany performed target hybridization, washing, staining and scanning after standard protocols of the manufacturer. For each growth condition, RNA from three independent cultures was used and three technical replicates were performed.

### Analysis of Expression Profiling Experiments

Raw data obtained from the Affymetrix GeneArray Scanner (e.g. CEL files) were preprocessed with the Bioconductor software [113]. Expression values were calculated using the Robust Multichip Average (RMA) method using quantile normalization, background corrected PM intensities and with median polish as summarization method [114], [115], [116]. In addition, the probabilities of differential expression (ppde) were computed for the pairwise comparison of experimental conditions of *P. aeruginosa* PAO1. We compared cells grown anaerobically for 2 days up to the late logarithmic phase on AUM supplemented with 50 mM nitrate versus 2 days on 10-fold diluted LB supplemented with 50 mM nitrate. All genes induced more than 2.0-fold and repressed more than 0.25-fold, respectively, and a ppde above 0.99999 were selected for further analysis. MIAME compliant array data were deposited in NCBI's Gene Expression Omnibus (Edgar et al., 2002) and are accessible through GEO-database accession number GSE33160 via the following link: http://www.ncbi.nlm.nih.gov/geo/info/linking.html.

### Quantitative Reverse Transcriptase PCR (qRT-PCR)

Quantitative reverse transcriptase PCR (qRT-PCR) was performed after the guidelines of Minimum Information for Publication of Quantitative (MIQE) [117]. Therefore, 1 ng and 10 ng of cDNA were used as a template. No-template and no-reverse transcription controls were carried out for each experiment. Primers were designed with Primer3Plus [118] using the following criteria: 100–150 bp product length, 18–30 bp primer length, 45–65% GC content, 63–67°C primer melting temperature and 65–85°C product melting temperature. Reference genes *gyrB* (PA0004) and *rpoD* (PA0567), constitutively expressed under the tested conditions were detected with the primer pairs: gyrB-fw `3-TTCGAGGTGGTGGATAAC-5′, gyrB-bw `3-GATATCCACCGGAATACC-5′ and rpoD-fw `3-AAGCGCAACAGCAATCTC-5′, rpoD-bw `3-GATGTCTTCCACCTGTTC-5′, respectively. For target genes following primers were used: *fur* (PA4764) fur-fw `3-GAGGTGATCGAGTTCATGGATGC-5′, fur-bw `3-GCACGTAGAGCACCAGATTGTGA-5′, *pvdS* (PA2426) pvdS-fw`3-GATAACCGTACGATCCTGGTGAAGA-5′, pvdS-bw`3-AGGTAGCTGAGCTGTGCCTTGAAC-5′, *lipA* (PA2862) lipA-fw `3-CAGCACCTACACCCAGACCAAATAC -5′, lipA-bw `3-GCTGACTTCGGTGACGTAGACCT-5′, *aprA* (PA1249) aprA-fw `3-ATATCTACTCGCTGGGCAAGTTCAG-5′, aprA-bw `3-GTCGACGAAGTGGATATTGGTGAC-5′, *lasB* (PA3724) lasB-fw `3-GACCAACACCTACAAGCAGGTCAAC-5′, lasB-bw `3-CTTCATGTACAGCTTGTGGGTCAG -5′, *narG* (PA3875) narG-fw `3-TGAACGGCACCAGCTTCTTC-5′, narG-bw `3-CGTTCGGCCTGGATGTTGTA-5′. qRT-PCR was performed with SsoFast EvaGreen Supermix (Bio-Rad, Munich, Germany) according to manufacturer's instructions and monitored with CFX96 Real-Time System (Bio-Rad, Munich, Germany). An initial denaturation step at 98°C for 3.5 min was followed by 40×repeated cycles of denaturation for 5 s at 98°C, primer annealing for 15 s at 59°C and elongation for 15 s at 60°C. Each cycle was followed by a plate read. Melting curves were generated by a final denaturation step for 10 s at 98°C and recorded within the range of 65–98°C. A slope of 3.3 was adjusted. Gene expression studies from the obtained data were calculated with CFX Manager V1.1 software (Bio-Rad, Munich, Germany). RNA from three biological replicas each were analysed and three technical replicates were performed.

### Proteome Analysis

A protocol previously described by Hanna and coworkers [119] was modified for cell fractionation and phenol extraction of proteins as indicated here. Proteins were extracted directly from *P. aeruginosa* cells with phenol and a subsequent acetone precipitation. The precipitated proteins were solubilized in sample buffer consisting of 7 M urea, 2 M thiourea, 4% (w/v) 3-((3- cholamidopropyl)-dimethylammonio)-1-propanesulfonate (CHAPS), 50 mM dithioerythritol (DTT), 2% (v/v) Triton X100 and 2% (w/v) ampholytes (Bio-Lyte; Biorad, Munich, Germany). Protein concentration was determined in the sample buffer using the 2-D Quant kit (Amersham, Freiburg, Germany).

Proteome analyses via 2-D gel electrophoresis with subsequently MALDI-TOF analysis were performed as described before [120]. 2-D electrophoresis was performed using immobilized pH gradient (IPG) strips of 17 cm length covering two pH ranges (pH 5–8 and pH 3–10; Biorad, Munich, Germany). A total of 350 µg protein was applied per gel. The gels were stained with Ruthenium II bathophenanthroline disulfonate chelate (RuBPS) [121] and subsequently documented with an FX-Scanner (Biorad, Munich, Germany). Analysis and quantification of differential protein spot patterns was performed using the Software Delta2D (Decodon, Greifswald, Germany).

Proteins of interest were excised and treated using a method of Shevchenko *et al.*, 2000 [122]. Proteins were identified by peptide-mass fingerprint (PMF) as well as post-source decay fragmentation data recorded on a Bruker Ultraflex MALDI-TOF mass spectrometer. PMF-data were analyzed using an internal MASCOT-server at the Helmholtz Centre Braunschweig (version 1.9; Matrix Science) [123] and the NCBI database restricted to the taxon *P. aeruginosa*. Only peptides with a MASCOT rank of 1 were considered significant and used for the combined peptide score. The criteria used to accept protein identifications based on PMF-data included extend of sequence coverage (minimum of 30%), the number of peptides matched (minimum of 5) and the score of probability (minimum of 70 for the Mowse score). Lower-scoring proteins were either verified manually or rejected.

### Metabolome Analysis

Cells were grown into the late logarithmic phase and harvested at the indicated time point. For each condition five cultures were grown and processed in parallel. For the extraction of the metabolites 150 mg wet weight *P. aeruginosa* cells were centrifuged at 8,000×g for 20 min at 4°C and washed three times with 2 ml 0.9% (w/v) NaCl at 10,000×g for 2 min at 4°C. Cells were mixed with 1.5 ml methanol and 7.5 µl ribitol (200 µg/ml as internal standard) and disrupted by freezing at −80°C and thawing three times. After centrifugation at 10,000×g for 2 min at 4°C, the supernatant was kept and the cells were resuspended in 1.0 ml water and centrifuged again. Resulting supernatants were pooled and 1.0 ml chloroform was added. The methanol/water phase and the chloroform phase were separated and samples dried overnight at RT in a rotary evaporator. For derivatization of the metabolites, the dry samples of the methanol/water phase were treated with 50 µl methoxyamine hydrochloride in pyridine (20 mg/ml) for 90 min at 30°C. The metabolites were subsequently trimethylsilylated using 80 µl MSTFA (N-Methyl-N-(trimethylsilyl)-trifluoroacetamide) for 30 min at 37°C and 2 h at RT. Subsequently, 10 µl of a standard was added, which contained alkanes of 10, 12, 15, 19, 22, 28, 32 and 36 carbon chain length (2 mg/ml each). Gaschromatography mass spectrometry (GC-MS) analysis has been carried out as described before [124] with a Finnigan Trace gas chromatograph carrying an AS 2000 autosampler and a Finnigan Trace mass spectrometer (Thermo Scientic, Massachusetts, USA). Metabolites were identified using the programs Xcalibur (Thermo Scientic, Massachusetts, USA) and AMDIS [125].

The obtained data were normalized via the retension time (Ri) of the standard alkanes and by dividing the intensity of each metabolome by the intensity of ribitol, which was added to each sample prior GC-MS measurements as internal standard. Afterwards, the data were processed statistically using a noise model integrated likelihood ratio test. A fold change cut-off of two and a *p-*value of <10^−5^ were applied. The metabolomes obtained for the various analysed conditions were compared using an emergent self-organizing map algorithm (ESOMet) [126]. Thereby, the metabolomics data of each sample was normalized to have a mean of zero and a standard deviation of 1 (Z-score normalization).

### Analysis of Enzyme Activities

Extracellular enzyme activities were determined photometrically in cell-free *P. aeruginosa* culture supernatants. For this purpose, biofilm cells grown to the late logarithmic phase were suspended in 0.9% (w/v) NaCl solution and subsequently sedimented by centrifugation. The supernatants were filtered through cellulose acetate membranes filters (0.2 mm pore-size). Lipase activity was measured with para-nitrophenyl-palmitate (pNPP) as a substrate. An absorbance at 410 nm of 1.0 in 15 min was related to a lipase activity of 48.3 nmol/min×ml supernatant [54]. Protease activity was determined using azocasein as a substrate. One enzyme unit of extracellular protease was defined as an increase of absorbance at 430 nm of 1.0 in 60 min [127].

### Analytical Methods

Uronic acid (alginate) concentrations in cell-free culture supernatants were determined with the biphenyl method [128] using isolated alginate from the mucoid *P. aeruginosa* strain FRD1 [129] as a standard. The alginate isolation procedure was performed as described before [130]. Protein concentrations were determined with the total protein kit, Micro-Lowry (Sigma-Aldrich, Steinheim, Germany) using bovine serum albumin (fraction V, Sigma-Aldrich, Steinheim, Germany) as a standard.

### Quorum Sensing Assay

The Acyl-homoserine lactones (AHL) were detected by an *Agrobacterium tumefaciens* based bioassay [131]. *A. tumefaciens*-glucose (ATG)-minimal medium agar plates containing 20 g/l NH_4_Cl, 6 g/l MgSO_4_×7 H_2_O, 3 g/l KCl, 200 mg/l CaCl_2_, 50 mg/l FeSO_4_×7 H_2_O, 60 g/l K_2_HPO_4_, 23 g/l NaH_2_PO_4_, 10% (w/v) D-glucose and 1.5% (w/v) agar were overlaid with 5 ml overnight culture of *A. tumefaciens* NTL4 (pZLR4) in 0.7% (w/v) ATG-soft agar containing 40 µg 5-bromo-4-chloro-3-indolyl-ß-D-galactopyranosid (X-Gal) and 0.7% (w/v) agar. Afterwards, wells (diameter of 0.5 cm) were punched in the agar plates and were filled with 50 µl cell-free *P. aeruginosa* culture supernatants. The plates were incubated overnight at 30°C. Blue halos around the wells indicate a positive reaction, which diameters were measured for quantification.

### Bioinformatic Approach

Data from transcriptome, proteome and metabolome analysis were stored and analyzed using the database SYSTOMONAS (www.systomonas.de; [132]), which integrated PRODORIC [58] as well as Kyoto Encyclopedia of Genes and Genomes (KEGG; [133], [134]) and is closely connected to BRENDA, the BRaunschweiger ENzyme Databank (www.brenda-enzymes.org; [135]. For visualisation of regulatory networks ProdoNet (www.prodonet.tu-bs.de; [59]) was used.

## Supporting Information

Figure S1 **Growth curves of** ***P. aeruginosa*** **PAO1** **under anaerobic conditions**. Colony biofilms were grown anaerobically on (▪) AUM or (□) 10-fold diluted LB agar at 37°C. Optical density was measured in biofilm suspensions. Results are expressed as mean value +/− standard derivation of three independent experiments performed in duplicates.
(JPG)

Table S1 **Differently expressed genes of** ***P. aeruginosa*** **PAO1.** The bacterium was grown anaerobically as biofilm up to the late logarithmic phase Pairwise comparisons of transcriptome data of AUM and 10-fold diluted LB grown biofilms were performed. To sustain anaerobic growth both media were supplemented with 50 mM potassium nitrate. A fold change cutoff of two and a ppde above 0.99999 were applied.
(DOCX)

Table S2 **Differently expressed genes involved in regulation of adaptation of** ***P. aeruginosa*** **PAO1 to urinary tract conditions.** Pairwise comparisons between late logarithmic biofilms grown anaerobically on AUM and 10-fold diluted LB supplemented with 50 mM nitrate was performed. A fold change cut-off of two and a ppde above 0.99999 was applied.
(DOCX)

Table S3 **Differently expressed genes of** ***P. aeruginosa*** **PAO1 involved in central metabolism.** Pairwise comparison between AUM-grown and 10-fold diluted LB-grown biofilms were performed. A fold change cut-off of two and a ppde above 0.99999 was applied.
(DOCX)

Table S4 **Differently expressed genes of** ***P. aeruginosa*** **PAO1 involved in amino acid metabolism.** Pairwise comparison between AUM-grown and 10-fold diluted LB-grown biofilms were performed. A fold change cut-off of two and a ppde above 0.99999 was applied.
(DOCX)

Table S5 **Proteins varying in their cellular concentration in biofilm cells of** ***P. aeruginosa*** **PAO1.** The bacterium was grown up to the late logarithmic phase on AUM compared to 10-fold diluted LB observed via 2D gel electrophoresis and subsequently MALDI-TOF analyses. For growth under anaerobic conditions the media were supplemented with 50 mM nitrate. A fold change cutoff of 10.0 was applied.
(DOCX)

Table S6 **Metabolites varying in their cellular concentration in cells of** ***P. aeruginosa*** **PAO1.** The bacterium was grown as biofilm under anaerobic conditions up to the late logarithmic phase. Pairwise comparisons between biofilms grown with AUM supplemented with 50 mM nitrate and biofilms grown with 10-fold diluted LB supplemented with 50 mM nitrate were performed. A fold change cutoff of two and a *p*-value of <10^−5^ was applied.
(DOCX)
