# Supplementary figures and images for: Regulatory and Metabolic Networks for the Adaptation of Pseudomonas aeruginosa Biofilms to Urinary Tract-Like Conditions
Source: PLoS One. 2013 Aug 13;8(8):e71845. doi: 10.1371/journal.pone.0071845 (PMC3742457; doi:10.1371/journal.pone.0071845)

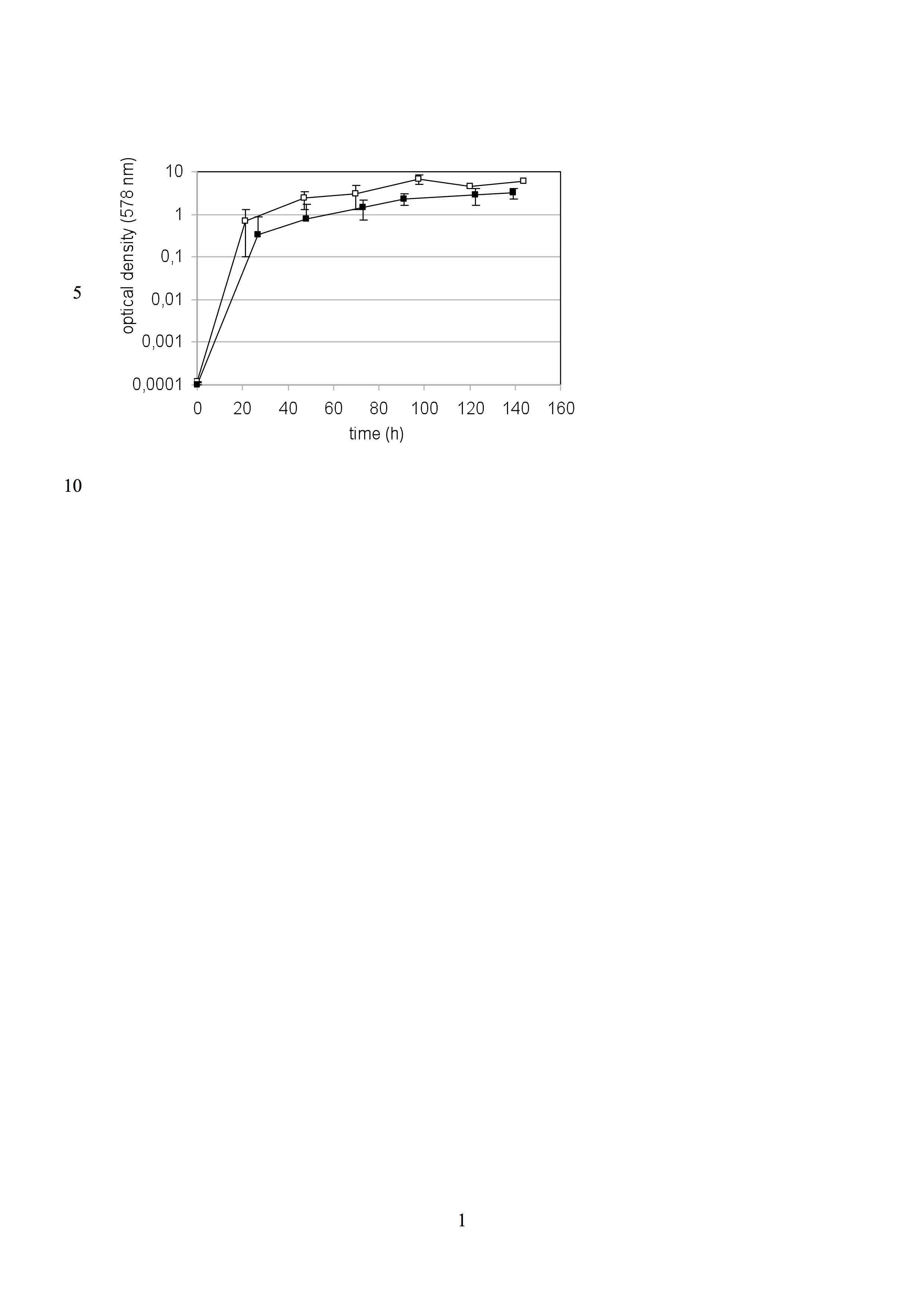

Supplement: Figure S1 — Growth curves of P. aeruginosa PAO1 under anaerobic conditions. Colony biofilms were grown anaerobically on (▪) AUM or (□) 10-fold diluted LB agar at 37°C. Optical density was measured in biofilm suspensions. Results are expressed as mean value +/− standard derivation of three independent experiments performed in duplicates. (JPG) [file pone.0071845.s001.jpg]
