# Supplementary material for: Regulatory and Metabolic Networks for the Adaptation of Pseudomonas aeruginosa Biofilms to Urinary Tract-Like Conditions
Source: PLoS One. 2013 Aug 13;8(8):e71845. doi: 10.1371/journal.pone.0071845 (PMC3742457; doi:10.1371/journal.pone.0071845)
Supplement: Table S1 — Differently expressed genes of P. aeruginosa PAO1. The bacterium was grown anaerobically as biofilm up to the late logarithmic phase Pairwise comparisons of transcriptome data of AUM and 10-fold diluted LB grown biofilms were performed. To sustain anaerobic growth both media were supplemented with 50 mM potassium nitrate. A fold change cutoff of two and a ppde above 0.99999 were applied. (DOCX) [file pone.0071845.s002.docx]

**Table S1.** **Differently expressed genes of *P. aeruginosa* PAO1.** The bacterium was grown anaerobically as biofilm up to the late logarithmic phase Pairwise comparisons of transcriptome data of AUM and 10-fold diluted LB grown biofilms were performed. To sustain anaerobic growth both media were supplemented with 50 mM potassium nitrate. A fold change cutoff of two and a ppde above 0.99999 were applied.

| Locus tag | Gene name | Product function | Fold change |
| --- | --- | --- | --- |
| PA0005 | *lptA* | lysophosphatidic acid acyltransferase, LptA | 0.45 |
| PA0023 | *qor* | NADPH:quinone reductase | 2.03 |
| PA0024 | *hemF* | coproporphyrinogen III oxidase, aerobic | 0.41 |
| PA0026 | *plcB* | phospholipase C PlcB | 0.38 |
| PA0035 | *trpA* | tryptophan synthase alpha chain | 0.42 |
| PA0036 | *trpB* | tryptophan synthase beta chain | 0.27 |
| PA0038 |  | hypothetical protein | 2.78 |
| PA0040 |  | conserved hypothetical protein | 0.46 |
| PA0045 |  | hypothetical protein | 0.41 |
| PA0049 |  | hypothetical protein | 3.41 |
| PA0059 | *osmC* | osmotically inducible protein OsmC | 3.36 |
| PA0070 |  | hypothetical protein | 0.35 |
| PA0072 |  | hypothetical protein | 0.36 |
| PA0073 |  | probable ATP-binding component of ABC transporter | 0.43 |
| PA0074 | *ppkA* | serine/threonine protein kinase PpkA | 0.29 |
| PA0075 |  | probable phosphoprotein phosphatase | 0.37 |
| PA0076 |  | hypothetical protein | 0.31 |
| PA0077 |  | hypothetical protein | 0.35 |
| PA0078 |  | hypothetical protein | 0.41 |
| PA0079 |  | hypothetical protein | 0.43 |
| PA0080 |  | hypothetical protein | 0.33 |
| PA0085 |  | conserved hypothetical protein | 0.48 |
| PA0086 |  | hypothetical protein | 0.36 |
| PA0087 |  | hypothetical protein | 0.28 |
| PA0088 |  | hypothetical protein | 0.29 |
| PA0089 |  | hypothetical protein | 0.27 |
| PA0090 |  | probable ClpA/B-type chaperone | 0.24 |
| PA0091 |  | conserved hypothetical protein | 0.22 |
| PA0109 |  | hypothetical protein | 3.12 |
| PA0122 |  | conserved hypothetical protein | 4.76 |
| PA0126 |  | hypothetical protein | 0.49 |
| PA0149 |  | probable sigma-70 factor, ECF subfamily | 2.07 |
| PA0160 |  | hypothetical protein | 0.16 |
| PA0180 |  | probable chemotaxis transducer | 0.41 |
| PA0183 | *atsA* | arylsulfatase | 2.87 |
| PA0184 | *atsC* | probable ATP-binding component of ABC transporter | 2.81 |
| PA0185 | *atsB* | probable permease of ABC transporter | 3.66 |
| PA0193 |  | hypothetical protein | 7.24 |
| PA0197 |  | hypothetical protein | 4.96 |
| PA0198 | *exbB1* | transport protein ExbB | 5.99 |
| PA0199 | *exbD1* | transport protein ExbD | 8.21 |
| PA0201 |  | hypothetical protein | 2.92 |
| PA0208 | *mdcA* | malonate decarboxylase alpha subunit | 0.35 |
| PA0209 | *mdcB* | conserved hypothetical protein | 0.25 |
| PA0210 | *mdcC* | malonate decarboxylase delta subunit | 0.16 |
| PA0211 | *mdcD* | malonate decarboxylase beta subunit | 0.22 |
| PA0212 | *mdcE* | malonate decarboxylase gamma subunit | 0.39 |
| PA0213 | *mdcG* | hypothetical protein | 0.23 |
| PA0214 | *mdcH* | probable acyl transferase | 0.21 |
| PA0215 | *madL* | probable transporter | 0.33 |
| PA0223 |  | probable dihydrodipicolinate synthetase | 0.46 |
| PA0224 |  | probable aldolase | 0.29 |
| PA0250 |  | conserved hypothetical protein | 2.05 |
| PA0269 |  | conserved hypothetical protein | 2.04 |
| PA0270 |  | hypothetical protein | 3.04 |
| PA0271 |  | hypothetical protein | 2.53 |
| PA0276 |  | hypothetical protein | 3.33 |
| PA0277 |  | conserved hypothetical protein | 0.37 |
| PA0281 | *cysW* | sulfate transport protein CysW | 0.42 |
| PA0291 | *oprE* | Anaerobically-induced outer membrane porin OprE precursor | 0.37 |
| PA0296 |  | probable glutamine synthetase | 3.97 |
| PA0297 | *spuA* | probable glutamine amidotransferase | 2.52 |
| PA0298 | *spuB* | probable glutamine synthetase | 3.72 |
| PA0299 | *spuC* | putrescine aminotransferase | 4.32 |
| PA0300 | *spuD* | polyamine transport protein | 2.68 |
| PA0307 |  | hypothetical protein | 0.48 |
| PA0315 |  | hypothetical protein | 3.93 |
| PA0316 | *serA* | D-3-phosphoglycerate dehydrogenase | 0.36 |
| PA0320 |  | conserved hypothetical protein | 0.31 |
| PA0347 | *glpQ* | glycerophosphoryl diester phosphodiesterase, periplasmic | 0.16 |
| PA0353 | *ilvD* | dihydroxy-acid dehydratase | 0.29 |
| PA0354 |  | conserved hypothetical protein | 0.49 |
| PA0380 |  | conserved hypothetical protein | 0.50 |
| PA0381 | *thiG* | thiamine biosynthesis protein, thiazole moiety | 0.49 |
| PA0382 | *micA* | DNA mismatch repair protein MicA | 0.27 |
| PA0386 | *yggW* | probable oxidase | 0.39 |
| PA0399 |  | cystathionine beta-synthase | 3.99 |
| PA0400 | *metC; metB* | probable cystathionine gamma-lyase | 3.98 |
| PA0422 |  | conserved hypothetical protein | 2.24 |
| PA0423 | *pasP* | PasP | 4.18 |
| PA0432 | *sahH* | S-adenosyl-L-homocysteine hydrolase | 0.49 |
| PA0433 |  | hypothetical protein | 4.41 |
| PA0434 |  | hypothetical protein | 2.22 |
| PA0449 |  | hypothetical protein | 2.70 |
| PA0459 | *clpC* | probable ClpA/B protease ATP binding subunit | 2.00 |
| PA0471 | *fiuR* | probable transmembrane sensor | 4.20 |
| PA0472 | *fiuI* | probable sigma-70 factor, ECF subfamily | 4.28 |
| PA0473 | *psfA* | probable glutathione S-transferase | 2.61 |
| PA0476 |  | probable permease | 3.57 |
| PA0478 |  | probable N-acetyltransferase | 0.24 |
| PA0483 |  | probable acetyltransferase | 2.08 |
| PA0484 |  | conserved hypothetical protein | 3.40 |
| PA0485 | *rarD* | conserved hypothetical protein | 0.32 |
| PA0501 | *bioF* | 8-amino-7-oxononanoate synthase | 0.44 |
| PA0509 | *nirN* | probable c-type cytochrome | 0.29 |
| PA0510 | *nirE* | probable uroporphyrin-III c-methyltransferase | 0.23 |
| PA0511 | *nirJ* | heme d1 biosynthesis protein NirJ | 0.29 |
| PA0512 | *nirH* | conserved hypothetical protein | 0.33 |
| PA0513 | *nirG* | probable transcriptional regulator | 0.17 |
| PA0514 | *nirL* | heme d1 biosynthesis protein NirL | 0.26 |
| PA0515 | *nirD* | probable transcriptional regulator | 0.17 |
| PA0516 | *nirF* | heme d1 biosynthesis protein NirF | 0.20 |
| PA0517 | *nirC* | probable c-type cytochrome precursor | 0.14 |
| PA0518 | *nirM* | cytochrome c-551 precursor | 0.12 |
| PA0519 | *nirS* | nitrite reductase precursor | 0.16 |
| PA0520 | *nirQ* | regulatory protein NirQ | 0.38 |
| PA0521 | *nirO* | probable cytochrome c oxidase subunit | 0.28 |
| PA0523 | *norC* | nitric-oxide reductase subunit C | 0.12 |
| PA0524 | *norB* | nitric-oxide reductase subunit B | 0.09 |
| PA0525 | *norD* | probable dinitrification protein NorD | 0.18 |
| PA0526 |  | hypothetical protein | 0.38 |
| PA0529 |  | conserved hypothetical protein | 0.16 |
| PA0530 |  | probable class III pyridoxal phosphate-dependent aminotransferase | 0.22 |
| PA0531 |  | probable glutamine amidotransferase | 0.18 |
| PA0541 |  | hypothetical protein | 0.28 |
| PA0548 | *tktA* | transketolase | 0.50 |
| PA0558 |  | conserved hypothetical protein | 2.02 |
| PA0567 | *yqaE* | conserved hypothetical protein | 2.15 |
| PA0578 |  | conserved hypothetical protein | 0.28 |
| PA0582 | *folB* | dihydroneopterin aldolase | 0.38 |
| PA0583 |  | hypothetical protein | 0.39 |
| PA0586 | *ycgB* | conserved hypothetical protein | 2.70 |
| PA0588 | *yeaG* | conserved hypothetical protein | 2.65 |
| PA0603 |  | probable ATP-binding component of ABC transporter | 2.21 |
| PA0604 |  | probable binding protein component of ABC transporter | 4.48 |
| PA0605 |  | probable permease of ABC transporter | 3.05 |
| PA0606 |  | probable permease of ABC transporter | 2.66 |
| PA0610 | *prtN* | transcriptional regulator PrtN | 4.09 |
| PA0612 | *ptrB* | repressor, PtrB | 4.05 |
| PA0613 |  | hypothetical protein | 3.29 |
| PA0614 |  | hypothetical protein | 2.91 |
| PA0615 |  | hypothetical protein | 3.22 |
| PA0616 |  | hypothetical protein | 2.56 |
| PA0617 |  | probable bacteriophage protein | 3.43 |
| PA0618 |  | probable bacteriophage protein | 3.48 |
| PA0619 |  | probable bacteriophage protein | 2.81 |
| PA0620 |  | probable bacteriophage protein | 3.02 |
| PA0621 |  | conserved hypothetical protein | 2.78 |
| PA0622 |  | probable bacteriophage protein | 2.97 |
| PA0623 |  | probable bacteriophage protein | 2.84 |
| PA0624 |  | hypothetical protein | 2.90 |
| PA0625 |  | hypothetical protein | 2.75 |
| PA0626 |  | hypothetical protein | 2.44 |
| PA0627 |  | conserved hypothetical protein | 3.89 |
| PA0628 |  | conserved hypothetical protein | 3.18 |
| PA0629 |  | conserved hypothetical protein | 3.10 |
| PA0630 |  | hypothetical protein | 3.33 |
| PA0631 |  | hypothetical protein | 2.08 |
| PA0632 |  | hypothetical protein | 4.31 |
| PA0633 |  | hypothetical protein | 2.53 |
| PA0634 |  | hypothetical protein | 2.62 |
| PA0635 |  | hypothetical protein | 3.21 |
| PA0636 |  | hypothetical protein | 3.25 |
| PA0637 |  | conserved hypothetical protein | 3.88 |
| PA0638 |  | probable bacteriophage protein | 2.95 |
| PA0639 |  | conserved hypothetical protein | 2.89 |
| PA0640 |  | probable bacteriophage protein | 3.11 |
| PA0641 |  | probable bacteriophage protein | 2.42 |
| PA0644 |  | hypothetical protein | 3.14 |
| PA0645 |  | hypothetical protein | 2.86 |
| PA0646 |  | hypothetical protein | 2.42 |
| PA0648 |  | hypothetical protein | 3.15 |
| PA0654 | *speD* | S-adenosylmethionine decarboxylase proenzyme | 0.20 |
| PA0656 | *ycfF* | probable HIT family protein | 2.28 |
| PA0672 | *hemO* | heme oxygenase | 7.36 |
| PA0674 | *pigC* | hypothetical protein | 0.10 |
| PA0675 |  | probable sigma-70 factor, ECF subfamily | 0.31 |
| PA0676 |  | probable transmembrane sensor | 0.41 |
| PA0677 | *hxcW* | HxcW putative pseudopilin | 0.39 |
| PA0679 | *hxcP* | hypothetical protein | 0.50 |
| PA0691 |  | hypothetical protein | 0.20 |
| PA0692 |  | hypothetical protein | 0.41 |
| PA0693 | *exbB2* | transport protein ExbB2 | 0.50 |
| PA0698 |  | hypothetical protein | 0.46 |
| PA0707 | *regA* | transcriptional regulator RegA | 7.40 |
| PA0730 |  | probable transferase | 0.20 |
| PA0734 |  | hypothetical protein | 0.48 |
| PA0740 | *sdsA1* | SDS hydrolase SdsA1 | 2.29 |
| PA0743 |  | probable 3-hydroxyisobutyrate dehydrogenase | 3.39 |
| PA0744 |  | probable enoyl-CoA hydratase/isomerase | 2.82 |
| PA0745 |  | probable enoyl-CoA hydratase/isomerase | 4.54 |
| PA0746 |  | probable acyl-CoA dehydrogenase | 2.78 |
| PA0747 |  | probable aldehyde dehydrogenase | 3.13 |
| PA0751 |  | conserved hypothetical protein | 8.81 |
| PA0752 |  | conserved hypothetical protein | 10.50 |
| PA0753 |  | hypothetical protein | 10.10 |
| PA0754 |  | hypothetical protein | 17.09 |
| PA0755 | *opdH* | cis-aconitate porin OpdH | 9.33 |
| PA0768 | *lepB* | signal peptidase I | 0.44 |
| PA0775 | *yecO* | conserved hypothetical protein | 0.49 |
| PA0782 | *putA* | proline dehydrogenase PutA | 4.40 |
| PA0783 | *putP* | sodium/proline symporter PutP | 4.11 |
| PA0792 | *prpD* | propionate catabolic protein PrpD | 4.63 |
| PA0795 | *prpC* | citrate synthase 2 | 3.11 |
| PA0805 |  | hypothetical protein | 2.14 |
| PA0807 | *ampDh3* | AmpDh3 | 8.57 |
| PA0820 |  | hypothetical protein | 2.28 |
| PA0842 |  | probable glycosyl transferase | 0.07 |
| PA0857 | *bolA* | morphogene protein BolA | 0.50 |
| PA0862 |  | hypothetical protein | 2.28 |
| PA0865 | *hpd* | 4-hydroxyphenylpyruvate dioxygenase | 5.39 |
| PA0866 | *aroP2* | aromatic amino acid transport protein AroP2 | 2.72 |
| PA0870 | *phhC* | aromatic amino acid aminotransferase | 4.16 |
| PA0871 | *phhB* | pterin-4-alpha-carbinolamine dehydratase | 4.14 |
| PA0872 | *phhA* | phenylalanine-4-hydroxylase | 2.43 |
| PA0874 |  | hypothetical protein | 0.23 |
| PA0887 | *acsA* | acetyl-coenzyme A synthetase | 6.40 |
| PA0888 | *aotJ* | arginine/ornithine binding protein AotJ | 0.30 |
| PA0892 | *aotP* | arginine/ornithine transport protein AotP | 0.36 |
| PA0904 | *lysC* | aspartate kinase alpha and beta chain | 0.36 |
| PA0907 |  | hypothetical protein | 2.06 |
| PA0909 |  | hypothetical protein | 2.52 |
| PA0910 |  | hypothetical protein | 6.02 |
| PA0911 |  | hypothetical protein | 4.09 |
| PA0915 | *yehS* | conserved hypothetical protein | 0.24 |
| PA0916 | *yliG* | conserved hypothetical protein | 0.36 |
| PA0929 | *pirR* | two-component response regulator | 3.56 |
| PA0931 | *pirA* | ferric enterobactin receptor PirA | 2.37 |
| PA0943 |  | hypothetical protein | 0.50 |
| PA0945 | *purM* | phosphoribosylaminoimidazole synthetase | 0.45 |
| PA0953 | *helX* | probable thioredoxin | 2.47 |
| PA0954 |  | probable acylphosphatase | 2.66 |
| PA0962 |  | probable dna-binding stress protein | 2.22 |
| PA0978 |  | conserved hypothetical protein | 0.46 |
| PA0979 |  | conserved hypothetical protein | 0.27 |
| PA0985 |  | pyocin S5 | 3.84 |
| PA0996 | *pqsA* | probable coenzyme A ligase | 2.30 |
| PA0997 | *pqsB* | Homologous to beta-keto-acyl-acyl-carrier protein synthase | 2.23 |
| PA0998 | *pqsC* | Homologous to beta-keto-acyl-acyl-carrier protein synthase | 2.21 |
| PA0999 | *pqsD* | 3-oxoacyl-[acyl-carrier-protein] synthase III | 2.01 |
| PA1001 | *phaA* | anthranilate synthase component I | 5.31 |
| PA1002 | *phaB* | anthranilate synthase component II | 4.38 |
| PA1003 | *mvfR; pqsR* | transcriptional regulator MvfR | 6.12 |
| PA1035 |  | hypothetical protein | 2.90 |
| PA1041 |  | probable outer membrane protein precursor | 2.05 |
| PA1053 | *slyB* | conserved hypothetical protein | 0.48 |
| PA1061 |  | conserved hypothetical protein | 0.34 |
| PA1070 | *braG* | branched-chain amino acid transport protein BraG | 2.38 |
| PA1071 | *braF* | branched-chain amino acid transport protein BraF | 2.01 |
| PA1074 | *braC* | branched-chain amino acid transport protein BraC | 4.02 |
| PA1077 | *flgB* | flagellar basal-body rod protein FlgB | 0.17 |
| PA1078 | *flgC* | flagellar basal-body rod protein FlgC | 0.22 |
| PA1079 | *flgD* | flagellar basal-body rod modification protein FlgD | 0.14 |
| PA1080 | *flgE* | flagellar hook protein FlgE | 0.16 |
| PA1081 | *flgF* | flagellar basal-body rod protein FlgF | 0.16 |
| PA1082 | *flgG* | flagellar basal-body rod protein FlgG | 0.26 |
| PA1083 | *flgH* | flagellar L-ring protein precursor FlgH | 0.26 |
| PA1084 | *flgI* | flagellar P-ring protein precursor FlgI | 0.28 |
| PA1085 | *flgJ* | flagellar protein FlgJ | 0.33 |
| PA1086 | *flgK* | flagellar hook-associated protein 1 FlgK | 0.19 |
| PA1087 | *flgL* | flagellar hook-associated protein type 3 FlgL | 0.23 |
| PA1088 |  | hypothetical protein | 0.26 |
| PA1089 |  | conserved hypothetical protein | 0.27 |
| PA1090 |  | hypothetical protein | 0.25 |
| PA1091 | *fgtA* | flagellar glycosyl transferase, FgtA | 0.26 |
| PA1092 | *fliC* | flagellin type B | 0.50 |
| PA1093 | *flaG* | hypothetical protein | 0.36 |
| PA1094 | *fliD* | flagellar capping protein FliD | 0.45 |
| PA1095 | *fliS* | hypothetical protein | 0.28 |
| PA1096 |  | hypothetical protein | 0.23 |
| PA1098 | *fleS* | two-component sensor | 0.38 |
| PA1099 | *fleR* | two-component response regulator | 0.39 |
| PA1100 | *fliE* | flagellar hook-basal body complex protein FliE | 0.36 |
| PA1101 | *fliF* | Flagella M-ring outer membrane protein precursor | 0.38 |
| PA1106 |  | hypothetical protein | 2.44 |
| PA1112 | *yliI* | conserved hypothetical protein | 2.25 |
| PA1116 |  | hypothetical protein | 0.40 |
| PA1122 | *fms ;pdf* | probable peptide deformylase | 2.05 |
| PA1134 |  | hypothetical protein | 2.69 |
| PA1135 | *yedU* | conserved hypothetical protein | 2.02 |
| PA1150 | *pys2* | pyocin S2 | 2.05 |
| PA1168 |  | hypothetical protein | 0.50 |
| PA1170 |  | conserved hypothetical protein | 0.42 |
| PA1175 | *napD* | NapD protein of periplasmic nitrate reductase | 2.94 |
| PA1176 | *napF* | ferredoxin protein NapF | 2.23 |
| PA1177 | *napE* | periplasmic nitrate reductase protein NapE | 4.95 |
| PA1189 |  | conserved hypothetical protein | 0.38 |
| PA1193 |  | hypothetical protein | 0.48 |
| PA1244 |  | hypothetical protein | 2.79 |
| PA1245 | *aprX* | hypothetical protein | 9.11 |
| PA1246 | *aprD* | alkaline protease secretion protein AprD | 3.40 |
| PA1247 | *aprE* | alkaline protease secretion protein AprE | 3.43 |
| PA1248 | *aprF* | Alkaline protease secretion outer membrane protein AprF precursor | 2.70 |
| PA1249 | *aprA* | alkaline metalloproteinase precursor | 24.69 |
| PA1288 | *ompP1* | probable outer membrane protein precursor | 3.82 |
| PA1300 |  | probable sigma-70 factor, ECF subfamily | 11.69 |
| PA1301 |  | probable transmembrane sensor | 10.13 |
| PA1317 | *cyoA* | cytochrome o ubiquinol oxidase subunit II | 0.05 |
| PA1318 | *cyoB* | cytochrome o ubiquinol oxidase subunit I | 0.09 |
| PA1319 | *cyoC* | cytochrome o ubiquinol oxidase subunit III | 0.13 |
| PA1320 | *cyoD* | cytochrome o ubiquinol oxidase subunit IV | 0.25 |
| PA1321 | *cyoE* | cytochrome o ubiquinol oxidase protein CyoE | 0.31 |
| PA1323 |  | hypothetical protein | 2.20 |
| PA1324 |  | hypothetical protein | 2.43 |
| PA1333 |  | hypothetical protein | 2.75 |
| PA1337 | *ansB* | glutaminase-asparaginase | 3.15 |
| PA1339 |  | probable ATP-binding component of ABC transporter | 3.84 |
| PA1340 |  | probable permease of ABC transporter | 2.45 |
| PA1341 |  | probable permease of ABC transporter | 2.15 |
| PA1342 |  | probable binding protein component of ABC transporter | 4.39 |
| PA1348 |  | hypothetical protein | 2.13 |
| PA1363 |  | probable sigma-70 factor, ECF subfamily | 2.70 |
| PA1365 |  | probable siderophore receptor | 2.09 |
| PA1376 | *aceK* | isocitrate dehydrogenase kinase/phosphatase | 2.01 |
| PA1377 | *yhhY* | conserved hypothetical protein | 2.17 |
| PA1404 |  | hypothetical protein | 2.53 |
| PA1409 | *aphA* | acetylpolyamine aminohydrolase | 0.22 |
| PA1423 |  | probable chemotaxis transducer | 0.47 |
| PA1428 | *yjaB* | conserved hypothetical protein | 0.48 |
| PA1441 |  | hypothetical protein | 0.27 |
| PA1442 | *fliL* | conserved hypothetical protein | 0.41 |
| PA1453 | *flhF* | flagellar biosynthesis protein FlhF | 0.46 |
| PA1456 | *cheY* | two-component response regulator CheY | 0.45 |
| PA1457 | *cheZ* | chemotaxis protein CheZ | 0.46 |
| PA1458 | *cheA* | probable two-component sensor | 0.34 |
| PA1459 | *cheB* | probable methyltransferase | 0.34 |
| PA1460 | *motC* | MotC | 0.49 |
| PA1461 | *motD* | MotD | 0.44 |
| PA1462 |  | probable plasmid partitioning protein | 0.40 |
| PA1463 |  | hypothetical protein | 0.35 |
| PA1464 | *cheW* | probable purine-binding chemotaxis protein | 0.46 |
| PA1465 |  | hypothetical protein | 0.40 |
| PA1471 |  | hypothetical protein | 0.21 |
| PA1473 |  | hypothetical protein | 0.32 |
| PA1474 |  | hypothetical protein | 0.44 |
| PA1478 | *helD ;cycX* | hypothetical protein | 0.27 |
| PA1480 | *ccmF* | cytochrome C-type biogenesis protein CcmF | 0.49 |
| PA1481 | *ccmG* | cytochrome C biogenesis protein CcmG | 0.45 |
| PA1482 | *ccmH* | cytochrome C-type biogenesis protein CcmH | 0.38 |
| PA1483 | *cycH* | cytochrome c-type biogenesis protein | 0.41 |
| PA1500 |  | probable oxidoreductase | 3.06 |
| PA1501 | *gip* | conserved hypothetical protein | 2.28 |
| PA1502 | *gcl* | glyoxylate carboligase | 2.83 |
| PA1507 |  | probable transporter | 5.73 |
| PA1513 |  | hypothetical protein | 4.35 |
| PA1514 | *ybbT* | conserved hypothetical protein | 2.63 |
| PA1515 | *alc* | allantoicase | 3.15 |
| PA1516 |  | hypothetical protein | 3.45 |
| PA1517 |  | conserved hypothetical protein | 5.33 |
| PA1518 |  | conserved hypothetical protein | 6.64 |
| PA1546 | *hemN* | oxygen-independent coproporphyrinogen III oxidase | 0.40 |
| PA1549 | *fixI* | probable cation-transporting P-type ATPase | 0.47 |
| PA1553 | *ccoO ;fixO* | probable cytochrome c oxidase subunit | 0.50 |
| PA1555 | *ccoP ;fixP* | probable cytochrome c | 0.43 |
| PA1556 | *ccoO ;fixO* | probable cytochrome c oxidase subunit | 0.35 |
| PA1557 | *ccoQ ;fixQ* | probable cytochrome c oxidase subunit | 0.50 |
| PA1571 |  | hypothetical protein | 0.43 |
| PA1572 |  | conserved hypothetical protein | 2.27 |
| PA1574 | *yaiE* | conserved hypothetical protein | 0.40 |
| PA1579 |  | hypothetical protein | 3.85 |
| PA1583 | *sdhA* | succinate dehydrogenase (A subunit) | 0.46 |
| PA1591 |  | hypothetical protein | 0.44 |
| PA1606 |  | hypothetical protein | 0.03 |
| PA1608 |  | probable chemotaxis transducer | 0.16 |
| PA1609 | *fabB* | beta-ketoacyl-ACP synthase I | 0.20 |
| PA1610 | *fabA* | beta-hydroxydecanoyl-ACP dehydrase | 0.20 |
| PA1612 |  | hypothetical protein | 0.47 |
| PA1617 |  | probable AMP-binding enzyme | 2.41 |
| PA1623 |  | conserved hypothetical protein | 2.29 |
| PA1651 |  | probable transporter | 0.31 |
| PA1677 |  | conserved hypothetical protein | 2.33 |
| PA1679 |  | hypothetical protein | 0.26 |
| PA1687 | *speE* | spermidine synthase | 0.49 |
| PA1688 |  | hypothetical protein | 0.39 |
| PA1689 |  | conserved hypothetical protein | 0.50 |
| PA1713 | *exsA* | transcriptional regulator ExsA | 2.09 |
| PA1728 |  | hypothetical protein | 3.30 |
| PA1745 |  | hypothetical protein | 2.62 |
| PA1746 |  | hypothetical protein | 7.35 |
| PA1757 | *thrH* | homoserine kinase | 0.49 |
| PA1760 |  | probable transcriptional regulator | 2.05 |
| PA1761 |  | hypothetical protein | 2.72 |
| PA1774 | *cfrX* | CfrX protein | 0.30 |
| PA1775 | *cmpX* | conserved cytoplasmic membrane protein, CmpX protein | 0.31 |
| PA1776 | *sigX* | ECF sigma factor SigX | 0.23 |
| PA1787 | *acnB* | aconitate hydratase 2 | 0.41 |
| PA1796 | *folD* | 5,10-methylene-tetrahydrofolate dehydrogenase / cyclohydrolase | 0.50 |
| PA1842 |  | hypothetical protein | 0.41 |
| PA1843 | *metH* | methionine synthase | 0.34 |
| PA1844 |  | hypothetical protein | 0.18 |
| PA1845 |  | hypothetical protein | 0.16 |
| PA1852 |  | hypothetical protein | 0.34 |
| PA1857 | *yedI* | conserved hypothetical protein | 0.48 |
| PA1862 | *modB* | molybdenum transport protein ModB | 0.37 |
| PA1863 | *modA* | molybdate-binding periplasmic protein precursor ModA | 0.09 |
| PA1864 |  | probable transcriptional regulator | 0.20 |
| PA1873 |  | hypothetical protein | 0.35 |
| PA1911 |  | probable transmembrane sensor | 6.79 |
| PA1912 |  | probable sigma-70 factor, ECF subfamily | 9.94 |
| PA1920 | *nrdD* | conserved hypothetical protein | 0.11 |
| PA1927 | *metE* | 5-methyltetrahydropteroyltriglutamate-homocysteine S-methyltransferase | 0.31 |
| PA1964 | *ybiT* | probable ATP-binding component of ABC transporter | 0.40 |
| PA1967 |  | hypothetical protein | 0.22 |
| PA1978 | *agmR* | probable transcriptional regulator | 2.33 |
| PA1984 | *exaC1* | probable aldehyde dehydrogenase | 3.58 |
| PA1985 | *pqqA* | pyrroloquinoline quinone biosynthesis protein A | 9.22 |
| PA1986 | *pqqB* | pyrroloquinoline quinone biosynthesis protein B | 3.55 |
| PA1987 | *pqqC* | pyrroloquinoline quinone biosynthesis protein C | 3.13 |
| PA1988 | *pqqD* | pyrroloquinoline quinone biosynthesis protein D | 3.97 |
| PA1989 | *pqqE* | pyrroloquinoline quinone biosynthesis protein E | 2.73 |
| PA2007 | *maiA* | maleylacetoacetate isomerase | 4.71 |
| PA2008 | *fahA* | fumarylacetoacetase | 3.82 |
| PA2009 | *hmgA* | homogentisate 1,2-dioxygenase | 3.92 |
| PA2014 | *liuB* | methylcrotonyl-CoA carboxylase, beta-subunit | 2.21 |
| PA2015 | *gnyD* | putative isovaleryl-CoA dehydrogenase | 3.82 |
| PA2016 | *gnyR* | regulator of liu genes GnyR | 2.63 |
| PA2022 |  | probable nucleotide sugar dehydrogenase | 0.50 |
| PA2023 | *galU* | UTP--glucose-1-phosphate uridylyltransferase | 0.46 |
| PA2024 |  | probable ring-cleaving dioxygenase | 3.61 |
| PA2026 | *yfeH* | conserved hypothetical protein | 0.41 |
| PA2033 |  | hypothetical protein | 20.89 |
| PA2034 |  | hypothetical protein | 8.95 |
| PA2052 | *cynS* | cyanate lyase | 2.46 |
| PA2062 |  | probable pyridoxal-phosphate dependent enzyme | 4.38 |
| PA2080 | *kynU* | hypothetical protein | 3.45 |
| PA2081 | *kynB* | kynurenine formamidase, KynB | 3.64 |
| PA2083 |  | probable ring-hydroxylating dioxygenase subunit | 24.41 |
| PA2084 | *yucB ;asnB* | probable asparagine synthetase | 5.10 |
| PA2085 |  | probable ring-hydroxylating dioxygenase small subunit | 10.58 |
| PA2086 |  | probable epoxide hydrolase | 9.01 |
| PA2087 |  | hypothetical protein | 6.35 |
| PA2088 |  | hypothetical protein | 12.79 |
| PA2089 |  | hypothetical protein | 3.37 |
| PA2090 |  | hypothetical protein | 9.76 |
| PA2091 |  | hypothetical protein | 3.97 |
| PA2092 |  | probable MFS transporter | 7.39 |
| PA2093 |  | probable sigma-70 factor, ECF subfamily | 6.28 |
| PA2094 |  | probable transmembrane sensor | 5.67 |
| PA2095 |  | hypothetical protein | 2.59 |
| PA2110 |  | hypothetical protein | 3.75 |
| PA2111 |  | hypothetical protein | 5.68 |
| PA2112 |  | conserved hypothetical protein | 6.50 |
| PA2113 | *opdO* | pyroglutamate porin OpdO | 5.38 |
| PA2114 |  | probable major facilitator superfamily (MFS) transporter | 12.30 |
| PA2116 |  | conserved hypothetical protein | 7.62 |
| PA2143 |  | hypothetical protein | 2.90 |
| PA2146 | *yciG* | conserved hypothetical protein | 21.24 |
| PA2169 |  | hypothetical protein | 2.49 |
| PA2173 |  | hypothetical protein | 2.17 |
| PA2184 | *yciE* | conserved hypothetical protein | 2.76 |
| PA2190 |  | conserved hypothetical protein | 7.93 |
| PA2193 | *hcnA* | hydrogen cyanide synthase HcnA | 0.47 |
| PA2194 | *hcnB* | hydrogen cyanide synthase HcnB | 0.40 |
| PA2195 | *hcnC* | hydrogen cyanide synthase HcnC | 0.46 |
| PA2204 |  | probable binding protein component of ABC transporter | 2.80 |
| PA2247 | *bkdA1* | 2-oxoisovalerate dehydrogenase (alpha subunit) | 2.55 |
| PA2248 | *bkdA2* | 2-oxoisovalerate dehydrogenase (beta subunit) | 2.91 |
| PA2249 | *bkdB* | branched-chain alpha-keto acid dehydrogenase | 2.15 |
| PA2250 | *lpdV* | lipoamide dehydrogenase-Val | 2.17 |
| PA2259 | *ptxS* | transcriptional regulator PtxS | 3.50 |
| PA2273 | *soxR* | probable transcriptional regulator | 2.08 |
| PA2274 |  | hypothetical protein | 0.50 |
| PA2277 | *arsR* | ArsR protein | 0.41 |
| PA2279 | *arsC* | ArsC protein | 0.21 |
| PA2285 |  | hypothetical protein | 0.39 |
| PA2305 |  | probable non-ribosomal peptide synthetase | 0.47 |
| PA2306 |  | conserved hypothetical protein | 0.16 |
| PA2327 |  | probable permease of ABC transporter | 2.60 |
| PA2328 |  | hypothetical protein | 2.43 |
| PA2364 |  | hypothetical protein | 2.26 |
| PA2366 |  | conserved hypothetical protein | 2.30 |
| PA2368 |  | hypothetical protein | 2.15 |
| PA2375 |  | hypothetical protein | 2.22 |
| PA2381 |  | hypothetical protein | 2.04 |
| PA2384 |  | hypothetical protein | 15.55 |
| PA2385 | *pvdQ* | PvdQ | 6.69 |
| PA2386 | *pvdA* | L-ornithine N5-oxygenase | 53.52 |
| PA2389 |  | conserved hypothetical protein | 6.10 |
| PA2390 |  | probable ATP-binding/permease fusion ABC transporter | 3.38 |
| PA2391 | *opmQ* | probable outer membrane protein precursor | 2.68 |
| PA2392 | *pvdP* | PvdP | 12.21 |
| PA2393 |  | probable dipeptidase precursor | 21.50 |
| PA2394 | *pvdN* | PvdN | 17.45 |
| PA2395 | *pvdO* | PvdO | 8.83 |
| PA2396 | *pvdF* | pyoverdine synthetase F | 12.35 |
| PA2397 | *pvdE* | pyoverdine biosynthesis protein PvdE | 14.79 |
| PA2398 | *fpvA* | ferripyoverdine receptor | 27.64 |
| PA2399 | *pvdD* | pyoverdine synthetase D | 7.22 |
| PA2400 | *pvdJ* | PvdJ | 8.11 |
| PA2401 |  |  | 6.59 |
| PA2402 |  | probable non-ribosomal peptide synthetase | 11.27 |
| PA2403 |  | hypothetical protein | 15.57 |
| PA2404 |  | hypothetical protein | 12.20 |
| PA2405 |  | hypothetical protein | 22.76 |
| PA2406 |  | hypothetical protein | 9.48 |
| PA2407 |  | probable adhesion protein | 6.38 |
| PA2408 |  | probable ATP-binding component of ABC transporter | 4.63 |
| PA2409 |  | probable permease of ABC transporter | 2.88 |
| PA2410 |  | hypothetical protein | 4.84 |
| PA2411 |  | probable thioesterase | 42.78 |
| PA2412 |  | conserved hypothetical protein | 70.23 |
| PA2413 | *pvdH* | L-2,4-diaminobutyrate:2-ketoglutarate 4-aminotransferase, PvdH | 33.26 |
| PA2424 | *pvdL* | PvdL | 13.32 |
| PA2425 | *pvdG* | PvdG | 8.95 |
| PA2426 | *pvdS* | sigma factor PvdS | 6.64 |
| PA2427 |  | hypothetical protein | 4.85 |
| PA2428 |  | hypothetical protein | 0.03 |
| PA2433 |  | hypothetical protein | 2.26 |
| PA2445 | *gcvP2* | glycine cleavage system protein P2 | 0.50 |
| PA2446 | *gcvH2* | glycine cleavage system protein H2 | 0.44 |
| PA2451 |  | hypothetical protein | 3.12 |
| PA2452 |  | hypothetical protein | 5.75 |
| PA2466 | *foxA* | Ferrioxamine receptor FoxA | 2.0 |
| PA2467 | *foxR* | Anti-sigma factor FoxR | 3.26 |
| PA2468 | *foxI* | ECF sigma factor FoxI | 4.30 |
| PA2483 | *yhbW* | conserved hypothetical protein | 3.31 |
| PA2531 |  | probable aminotransferase | 3.41 |
| PA2536 | *ynbB* | probable phosphatidate cytidylyltransferase | 0.47 |
| PA2537 |  | probable acyltransferase | 0.28 |
| PA2538 |  | hypothetical protein | 0.34 |
| PA2539 | *ynbD* | conserved hypothetical protein | 0.45 |
| PA2540 |  | conserved hypothetical protein | 0.33 |
| PA2548 |  | hypothetical protein | 0.20 |
| PA2551 |  | probable transcriptional regulator | 3.17 |
| PA2552 | *acdB* | probable acyl-CoA dehydrogenase | 2.09 |
| PA2553 |  | probable acyl-CoA thiolase | 2.82 |
| PA2561 |  | probable chemotaxis transducer | 0.28 |
| PA2568 |  | hypothetical protein | 2.22 |
| PA2572 |  | probable two-component response regulator | 2.02 |
| PA2573 |  | probable chemotaxis transducer | 2.51 |
| PA2584 | *pgsA* | CDP-diacylglycerol--glycerol-3-phosphate 3-phosphatidyltransferase | 0.41 |
| PA2590 |  | hypothetical protein | 2.31 |
| PA2604 | *yccA* | conserved hypothetical protein | 2.05 |
| PA2619 | *infA* | initiation factor | 0.48 |
| PA2622 | *cspD* | cold-shock protein CspD | 2.06 |
| PA2623 | *icd* | isocitrate dehydrogenase | 3.54 |
| PA2629 | *purB* | adenylosuccinate lyase | 0.26 |
| PA2630 | *ycfD* | conserved hypothetical protein | 0.24 |
| PA2635 |  | hypothetical protein | 0.11 |
| PA2637 | *nuoA* | NADH dehydrogenase I chain A | 0.43 |
| PA2638 | *nuoB* | NADH dehydrogenase I chain B | 0.38 |
| PA2641 | *nuoF* | NADH dehydrogenase I chain F | 0.49 |
| PA2642 | *nuoG* | NADH dehydrogenase I chain G | 0.43 |
| PA2643 | *nuoH* | NADH dehydrogenase I chain H | 0.45 |
| PA2645 | *nuoJ* | NADH dehydrogenase I chain J | 0.38 |
| PA2646 | *nuoK* | NADH dehydrogenase I chain K | 0.36 |
| PA2647 | *nuoL* | NADH dehydrogenase I chain L | 0.49 |
| PA2648 | *nuoM* | NADH dehydrogenase I chain M | 0.39 |
| PA2649 | *nuoN* | NADH dehydrogenase I chain N | 0.40 |
| PA2652 |  | probable chemotaxis transducer | 0.15 |
| PA2654 |  | probable chemotaxis transducer | 0.29 |
| PA2662 |  | conserved hypothetical protein | 0.29 |
| PA2663 |  | hypothetical protein | 0.25 |
| PA2664 | *fhp* | flavohemoprotein | 0.25 |
| PA2666 | *ptpS* | probable 6-pyruvoyl tetrahydrobiopterin synthase | 0.46 |
| PA2679 |  | hypothetical protein | 4.31 |
| PA2686 | *pfeR* | two-component response regulator PfeR | 3.12 |
| PA2688 | *pfeA* | Ferric enterobactin receptor, outer membrane protein PfeA | 2.50 |
| PA2716 |  | probable FMN oxidoreductase | 0.11 |
| PA2717 | *cpo* | chloroperoxidase precursor | 0.25 |
| PA2719 |  | hypothetical protein | 0.24 |
| PA2729 |  | hypothetical protein | 0.49 |
| PA2746 |  | hypothetical protein | 12.10 |
| PA2747 |  | hypothetical protein | 2.89 |
| PA2765 |  | hypothetical protein | 0.42 |
| PA2776 | *ordL* | conserved hypothetical protein | 3.03 |
| PA2786 |  | hypothetical protein | 2.56 |
| PA2788 |  | probable chemotaxis transducer | 0.21 |
| PA2796 | *tal* | dihydroxyacetone transferase | 2.13 |
| PA2803 |  | hypothetical protein | 0.06 |
| PA2804 |  | hypothetical protein | 0.05 |
| PA2817 |  | hypothetical protein | 0.46 |
| PA2840 | *deaD* | probable ATP-dependent RNA helicase | 0.08 |
| PA2850 | *ohr* | organic hydroperoxide resistance protein | 0.38 |
| PA2862 | *lipA* | triacylglycerol lipase | 8.83 |
| PA2863 | *lipH* | lipase modulator protein | 2.05 |
| PA2867 |  | probable chemotaxis transducer | 0.25 |
| PA2881 |  | probable two-component response regulator | 0.13 |
| PA2882 |  | probable two-component sensor | 0.16 |
| PA2883 |  | hypothetical protein | 3.64 |
| PA2901 |  | hypothetical protein | 0.50 |
| PA2915 |  | hypothetical protein | 2.36 |
| PA2929 |  | hypothetical protein | 4.57 |
| PA2937 |  | hypothetical protein | 4.64 |
| PA2950 |  | hypothetical protein | 0.30 |
| PA2953 |  | electron transfer flavoprotein-ubiquinone oxidoreductase | 0.47 |
| PA2954 |  | hypothetical protein | 0.49 |
| PA2957 |  | probable transcriptional regulator | 0.46 |
| PA2965 | *fabF1* | beta-ketoacyl-acyl carrier protein synthase II | 0.47 |
| PA2967 | *fabG* | 3-oxoacyl-[acyl-carrier-protein] reductase | 0.21 |
| PA2968 | *fabD* | malonyl-CoA-[acyl-carrier-protein] transacylase | 0.10 |
| PA2987 | *ycfV* | probable ATP-binding component of ABC transporter | 0.50 |
| PA3001 |  | probable glyceraldehyde-3-phosphate dehydrogenase | 0.47 |
| PA3014 | *faoA, fadB* | fatty-acid oxidation complex alpha-subunit | 0.40 |
| PA3038 | *opdQ* | probable porin | 2.02 |
| PA3041 | *yqjE* | hypothetical protein | 2.28 |
| PA3049 | *rmf* | ribosome modulation factor | 2.26 |
| PA3079 |  | hypothetical protein | 2.31 |
| PA3080 |  | hypothetical protein | 2.24 |
| PA3094 |  | probable transcriptional regulator | 2.08 |
| PA3106 |  | probable short-chain dehydrogenase | 0.39 |
| PA3108 | *purF* | amidophosphoribosyltransferase | 0.45 |
| PA3111 | *folC* | folylpolyglutamate synthetase | 0.46 |
| PA3112 | *accD* | acetyl-CoA carboxylase beta subunit | 0.42 |
| PA3117 | *asd* | aspartate semialdehyde dehydrogenase | 0.43 |
| PA3118 | *leuB* | 3-isopropylmalate dehydrogenase | 0.35 |
| PA3119 | *yafE* | conserved hypothetical protein | 0.46 |
| PA3123 |  | conserved hypothetical protein | 2.37 |
| PA3139 | *tyrB ;aspC* | probable amino acid aminotransferase | 0.35 |
| PA3149 | *wbpH* | probable glycosyltransferase WbpH | 0.49 |
| PA3161 | *himD* | integration host factor beta subunit | 2.09 |
| PA3195 | *gapA* | glyceraldehyde 3-phosphate dehydrogenase | 2.89 |
| PA3205 |  | hypothetical protein | 0.12 |
| PA3216 |  | hypothetical protein | 0.48 |
| PA3219 |  | hypothetical protein | 0.14 |
| PA3232 |  | probable nuclease | 3.83 |
| PA3233 |  | hypothetical protein | 5.93 |
| PA3234 | *yjcG* | probable sodium:solute symporter | 8.91 |
| PA3235 | *yjcH* | conserved hypothetical protein | 7.84 |
| PA3246 | *rluA* | pseudouridine synthase RluA | 0.42 |
| PA3249 |  | probable transcriptional regulator | 0.38 |
| PA3250 |  | hypothetical protein | 0.08 |
| PA3251 |  | hypothetical protein | 0.13 |
| PA3252 |  | probable permease of ABC transporter | 0.26 |
| PA3253 |  | probable permease of ABC transporter | 0.21 |
| PA3254 |  | probable ATP-binding component of ABC transporter | 0.21 |
| PA3255 |  | hypothetical protein | 0.31 |
| PA3258 |  | hypothetical protein | 0.10 |
| PA3271 |  | probable two-component sensor | 2.15 |
| PA3274 |  | hypothetical protein | 2.38 |
| PA3279 | *oprP* | Phosphate-specific outer membrane porin OprP precursor | 0.31 |
| PA3280 | *oprO* | Pyrophosphate-specific outer membrane porin OprO precursor | 0.01 |
| PA3296 | *phoA* | alkaline phosphatase | 0.05 |
| PA3307 |  | hypothetical protein | 0.49 |
| PA3308 | *hepA* | RNA helicase HepA | 0.43 |
| PA3319 | *plcN* | non-hemolytic phospholipase C precursor | 0.19 |
| PA3326 |  | probable Clp-family ATP-dependent protease | 2.31 |
| PA3349 |  | probable chemotaxis protein | 0.32 |
| PA3351 | *flgM* | FlgM | 0.45 |
| PA3352 |  | hypothetical protein | 0.44 |
| PA3356 |  | conserved hypothetical protein | 3.15 |
| PA3361 | *lecB* | fucose-binding lectin PA-IIL | 2.33 |
| PA3368 |  | probable acetyltransferase | 0.13 |
| PA3369 |  | hypothetical protein | 0.23 |
| PA3370 |  | hypothetical protein | 0.31 |
| PA3371 |  | hypothetical protein | 0.28 |
| PA3375 | *phnL* | probable ATP-binding component of ABC transporter | 0.27 |
| PA3376 | *phnK* | probable ATP-binding component of ABC transporter | 0.32 |
| PA3377 | *phnJ* | conserved hypothetical protein | 0.11 |
| PA3378 | *phnI* | conserved hypothetical protein | 0.13 |
| PA3379 | *phnH* | conserved hypothetical protein | 0.18 |
| PA3380 | *phnG* | conserved hypothetical protein | 0.14 |
| PA3381 | *phnF* | probable transcriptional regulator | 0.11 |
| PA3382 | *phnE* | phosphonate transport protein PhnE | 0.23 |
| PA3383 | *phnD* | binding protein component of ABC phosphonate transporter | 0.05 |
| PA3384 | *phnC* | ATP-binding component of ABC phosphonate transporter | 0.15 |
| PA3391 | *nosR* | regulatory protein NosR | 0.22 |
| PA3392 | *nosZ* | nitrous-oxide reductase precursor | 0.09 |
| PA3393 | *nosD* | NosD protein | 0.21 |
| PA3394 | *nosF* | NosF protein | 0.21 |
| PA3395 | *nosY* | NosY protein | 0.34 |
| PA3396 | *nosL* | NosL protein | 0.40 |
| PA3399 |  | hypothetical protein | 2.02 |
| PA3405 | *hasE* | metalloprotease secretion protein | 2.43 |
| PA3406 | *hasD* | transport protein HasD | 3.18 |
| PA3407 | *hasAp* | heme acquisition protein HasAp | 63.97 |
| PA3408 | *hasR* | Haem uptake outer membrane receptor HasR precursor | 10.00 |
| PA3409 |  | probable transmembrane sensor | 3.60 |
| PA3410 |  | probable sigma-70 factor, ECF subfamily | 8.41 |
| PA3415 |  | probable dihydrolipoamide acetyltransferase | 2.14 |
| PA3416 |  | probable pyruvate dehydrogenase E1 component, beta chain | 2.15 |
| PA3417 |  | probable pyruvate dehydrogenase E1 component, alpha subunit | 2.28 |
| PA3418 | *ldh* | leucine dehydrogenase | 3.04 |
| PA3441 | *ssuF* | probable molybdopterin-binding protein | 8.10 |
| PA3450 | *lsfA* | probable antioxidant protein | 3.16 |
| PA3479 | *rhlA* | rhamnosyltransferase chain A | 2.39 |
| PA3510 |  | hypothetical protein | 2.13 |
| PA3520 |  | hypothetical protein | 5.46 |
| PA3525 | *argG* | argininosuccinate synthase | 0.45 |
| PA3526 | *motY* | probable outer membrane protein precursor | 0.18 |
| PA3530 | *bfd* | bacterioferritin-associated ferredoxin | 5.03 |
| PA3531 | *bfrB* | bacterioferritin | 0.27 |
| PA3568 | *ymmS* | probable acetyl-coa synthetase | 5.83 |
| PA3569 | *mmsB* | 3-hydroxyisobutyrate dehydrogenase | 2.75 |
| PA3570 | *mmsA* | methylmalonate-semialdehyde dehydrogenase | 3.06 |
| PA3580 | *ybaK* | conserved hypothetical protein | 0.42 |
| PA3584 | *glpD* | glycerol-3-phosphate dehydrogenase | 0.10 |
| PA3600 | *rpl36* | conserved hypothetical protein | 32.08 |
| PA3601 | *ykgM* | conserved hypothetical protein | 25.36 |
| PA3602 | *yerD* | conserved hypothetical protein | 0.22 |
| PA3603 | *dgkA* | diacylglycerol kinase | 0.50 |
| PA3614 |  | hypothetical protein | 2.38 |
| PA3621 | *fdxA* | ferredoxin I | 0.40 |
| PA3632 | *yedF* | conserved hypothetical protein | 0.44 |
| PA3633 | *ygbP* | 4-diphosphocytidyl-2-C-methylerythritol synthase | 0.41 |
| PA3639 | *accA* | acetyl-coenzyme A carboxylase carboxyl transferase (alpha subunit) | 0.48 |
| PA3642 | *rnhB* | ribonuclease HII | 0.45 |
| PA3643 | *lpxB* | lipid A-disaccharide synthase | 0.35 |
| PA3645 | *fabZ* | (3R)-hydroxymyristoyl-[acyl carrier protein] dehydratase | 0.27 |
| PA3653 | *frr* | ribosome recycling factor | 0.50 |
| PA3654 | *pyrH* | uridylate kinase | 0.41 |
| PA3659 | *dapC* | probable aminotransferase | 0.49 |
| PA3661 |  | hypothetical protein | 0.15 |
| PA3688 |  | hypothetical protein | 2.49 |
| PA3690 |  | probable metal-transporting P-type ATPase | 0.38 |
| PA3691 |  | hypothetical protein | 3.00 |
| PA3692 |  | probable outer membrane protein precursor | 3.86 |
| PA3700 | *lysS* | lysyl-tRNA synthetase | 0.40 |
| PA3712 |  | hypothetical protein | 3.27 |
| PA3723 | *yqjM* | probable FMN oxidoreductase | 2.15 |
| PA3724 | *lasB* | elastase LasB | 4.77 |
| PA3727 |  | hypothetical protein | 0.35 |
| PA3728 |  | hypothetical protein | 0.40 |
| PA3741 |  | hypothetical protein | 0.24 |
| PA3743 | *trmD* | tRNA (guanine-N1)-methyltransferase | 0.44 |
| PA3744 | *rimM* | 16S rRNA processing protein | 0.48 |
| PA3745 | *rpsP* | 30S ribosomal protein S16 | 0.44 |
| PA3749 | *yhjE* | probable major facilitator superfamily (MFS) transporter | 0.37 |
| PA3756 | *yafK* | hypothetical protein | 0.50 |
| PA3769 | *guaA* | GMP synthase | 0.29 |
| PA3770 | *guaB* | inosine-5'-monophosphate dehydrogenase | 0.34 |
| PA3779 |  | hypothetical protein | 2.15 |
| PA3784 |  | hypothetical protein | 2.27 |
| PA3799 | *yfgK* | conserved hypothetical protein | 0.50 |
| PA3802 | *hisS* | histidyl-tRNA synthetase | 0.49 |
| PA3807 | *ndk* | nucleoside diphosphate kinase | 0.42 |
| PA3808 | *yfhJ* | conserved hypothetical protein | 0.48 |
| PA3818 | *suhB* | extragenic suppressor protein SuhB | 0.30 |
| PA3823 | *tgt* | queuine tRNA-ribosyltransferase | 0.44 |
| PA3824 | *queA* | S-adenosylmethionine:trna ribosyltransferase-isomerase | 0.42 |
| PA3827 | *yjgQ* | conserved hypothetical protein | 0.49 |
| PA3836 |  | hypothetical protein | 2.63 |
| PA3846 |  | hypothetical protein | 2.34 |
| PA3866 |  | pyocin protein | 2.98 |
| PA3871 | *nifM* | probable peptidyl-prolyl cis-trans isomerase, PpiC-type | 0.33 |
| PA3872 | *narI* | respiratory nitrate reductase gamma chain | 0.19 |
| PA3873 | *narJ* | respiratory nitrate reductase delta chain | 0.21 |
| PA3874 | *narH* | respiratory nitrate reductase beta chain | 0.13 |
| PA3875 | *narG* | respiratory nitrate reductase alpha chain | 0.15 |
| PA3876 | *narK2* | nitrite extrusion protein 2 | 0.50 |
| PA3877 | *narK1* | nitrite extrusion protein 1 | 0.23 |
| PA3878 | *narX* | two-component sensor NarX | 2.67 |
| PA3879 | *narL* | two-component response regulator NarL | 2.47 |
| PA3880 |  | conserved hypothetical protein | 0.23 |
| PA3899 |  | probable sigma-70 factor, ECF subfamily | 7.12 |
| PA3900 |  | probable transmembrane sensor | 3.24 |
| PA3901 | *fecA* | Fe(III) dicitrate transport protein FecA | 17.08 |
| PA3904 |  | hypothetical protein | 0.31 |
| PA3905 |  | hypothetical protein | 0.43 |
| PA3906 |  | hypothetical protein | 0.29 |
| PA3907 |  | hypothetical protein | 0.36 |
| PA3908 |  | hypothetical protein | 0.34 |
| PA3909 |  | hypothetical protein | 0.16 |
| PA3910 |  | hypothetical protein | 0.14 |
| PA3911 | *yhbT* | conserved hypothetical protein | 0.18 |
| PA3912 | *yhbV* | conserved hypothetical protein | 0.25 |
| PA3913 | *yhbU* | probable protease | 0.13 |
| PA3914 | *moeA1* | molybdenum cofactor biosynthetic protein A1 | 0.16 |
| PA3915 | *moaB1* | molybdopterin biosynthetic protein B1 | 0.05 |
| PA3916 | *moaE* | molybdopterin converting factor, large subunit | 0.33 |
| PA3917 | *moaD* | molybdopterin converting factor, small subunit | 0.38 |
| PA3918 | *moaC* | molybdopterin biosynthetic protein C | 0.24 |
| PA3921 |  | probable transcriptional regulator | 3.21 |
| PA3922 |  | conserved hypothetical protein | 7.98 |
| PA3923 |  | hypothetical protein | 8.49 |
| PA3924 |  | probable medium-chain acyl-CoA ligase | 2.46 |
| PA3925 |  | probable acyl-CoA thiolase | 2.17 |
| PA3928 |  | hypothetical protein | 0.18 |
| PA3929 | *cioB* | cyanide insensitive terminal oxidase | 0.32 |
| PA3930 | *cioA* | cyanide insensitive terminal oxidase | 0.14 |
| PA3967 |  | hypothetical protein | 0.32 |
| PA3979 |  | hypothetical protein | 0.31 |
| PA3986 |  | hypothetical protein | 2.08 |
| PA3990 |  | conserved hypothetical protein | 0.42 |
| PA4021 |  | probable transcriptional regulator | 2.79 |
| PA4023 | *eutP* | probable transport protein | 9.65 |
| PA4024 | *eutB* | ethanolamine ammonia-lyase large subunit | 8.83 |
| PA4025 | *eutC* | probable ethanolamine ammonia-lyase light chain | 4.57 |
| PA4044 | *dxs* | 1-deoxyxylulose-5-phosphate synthase | 0.49 |
| PA4054 | *ribB* | GTP cyclohydrolase II / 3,4-dihydroxy-2-butanone 4-phosphate synthase | 0.39 |
| PA4055 | *ribC* | riboflavin synthase alpha chain | 0.33 |
| PA4063 |  | hypothetical protein | 2.17 |
| PA4067 | *oprG* | Outer membrane protein OprG precursor | 0.48 |
| PA4090 |  | hypothetical protein | 2.58 |
| PA4129 |  | hypothetical protein | 0.17 |
| PA4130 |  | probable sulfite or nitrite reductase | 0.17 |
| PA4131 |  | probable iron-sulfur protein | 0.09 |
| PA4132 |  | conserved hypothetical protein | 0.17 |
| PA4133 | *ccoN; fixN* | cytochrome c oxidase subunit (cbb3-type) | 0.10 |
| PA4134 |  | hypothetical protein | 0.32 |
| PA4139 |  | hypothetical protein | 0.10 |
| PA4141 |  | hypothetical protein | 3.77 |
| PA4156 |  | probable TonB-dependent receptor | 2.71 |
| PA4168 | *fpvB* | second ferric pyoverdine receptor FpvB | 5.04 |
| PA4171 |  | probable protease | 2.74 |
| PA4207 | *mexI* | probable Resistance-Nodulation-Cell Division (RND) efflux transporter | 0.49 |
| PA4218 |  | probable transporter | 3.32 |
| PA4219 | *yfpB* | hypothetical protein | 4.58 |
| PA4220 | *fptB* | hypothetical protein | 4.01 |
| PA4221 | *fptA* | Fe(III)-pyochelin outer membrane receptor precursor | 3.18 |
| PA4222 | *pchI* | probable ATP-binding component of ABC transporter | 7.24 |
| PA4223 | *pchH* | probable ATP-binding component of ABC transporter | 6.32 |
| PA4224 | *pchG* | pyochelin biosynthetic protein PchG | 7.12 |
| PA4225 | *pchF* | pyochelin synthetase | 8.89 |
| PA4226 | *pchE* | dihydroaeruginoic acid synthetase | 8.14 |
| PA4227 | *pchR* | transcriptional regulator PchR | 4.48 |
| PA4228 | *pchD* | pyochelin biosynthesis protein PchD | 3.57 |
| PA4229 | *pchC* | pyochelin biosynthetic protein PchC | 3.68 |
| PA4230 | *pchB* | chorismate mutase PchB | 5.18 |
| PA4231 | *pchA* | salicylate biosynthesis isochorismate synthase | 3.90 |
| PA4235 | *bfrA* | bacterioferritin | 0.37 |
| PA4239 | *rpsD* | 30S ribosomal protein S4 | 0.50 |
| PA4266 | *fusA1* | elongation factor G | 0.45 |
| PA4269 | *rpoC* | DNA-directed RNA polymerase beta* chain | 0.34 |
| PA4270 | *rpoB* | DNA-directed RNA polymerase beta chain | 0.39 |
| PA4273 | *rplA* | 50S ribosomal protein L1 | 0.50 |
| PA4275 | *nusG* | transcription antitermination protein NusG | 0.43 |
| PA4276 | *secE* | secretion protein SecE | 0.41 |
| PA4289 |  | probable transporter | 2.03 |
| PA4290 |  | probable chemotaxis transducer | 7.19 |
| PA4292 |  | probable phosphate transporter | 0.36 |
| PA4296 | *pprB* | two-component response regulator, PprB | 6.87 |
| PA4309 | *pctA* | chemotactic transducer PctA | 0.17 |
| PA4310 | *pctB* | chemotactic transducer PctB | 0.17 |
| PA4311 |  | conserved hypothetical protein | 2.06 |
| PA4318 |  | hypothetical protein | 0.48 |
| PA4321 |  | hypothetical protein | 0.49 |
| PA4322 |  | conserved hypothetical protein | 0.49 |
| PA4323 |  | hypothetical protein | 0.46 |
| PA4326 |  | hypothetical protein | 0.21 |
| PA4333 | *fumA* | probable fumarase | 0.27 |
| PA4340 |  | hypothetical protein | 2.08 |
| PA4348 |  | conserved hypothetical protein | 0.37 |
| PA4350 |  | conserved hypothetical protein | 0.05 |
| PA4351 |  | probable acyltransferase | 0.05 |
| PA4354 |  | conserved hypothetical protein | 2.20 |
| PA4359 | *feoA* | conserved hypothetical protein | 0.36 |
| PA4370 | *icmP* | Insulin-cleaving metalloproteinase outer membrane protein precursor | 2.84 |
| PA4377 |  | hypothetical protein | 2.77 |
| PA4385 | *groEL* | GroEL protein | 2.73 |
| PA4386 | *groES* | GroES protein | 2.64 |
| PA4428 | *sspA* | stringent starvation protein A | 0.47 |
| PA4429 |  | probable cytochrome c1 precursor | 0.25 |
| PA4430 |  | probable cytochrome b | 0.27 |
| PA4431 |  | probable iron-sulfur protein | 0.48 |
| PA4432 | *rpsI* | 30S ribosomal protein S9 | 0.39 |
| PA4438 | *yhcM* | conserved hypothetical protein | 0.45 |
| PA4441 |  | hypothetical protein | 0.39 |
| PA4467 |  | hypothetical protein | 12.28 |
| PA4468 | *sodM* | superoxide dismutase | 19.81 |
| PA4469 |  | hypothetical protein | 22.10 |
| PA4470 | *fumC1* | fumarate hydratase | 18.92 |
| PA4471 | *fagA* | hypothetical protein | 18.91 |
| PA4479 | *mreD* | rod shape-determining protein MreD | 0.44 |
| PA4480 | *mreC* | rod shape-determining protein MreC | 0.44 |
| PA4487 |  | conserved hypothetical protein | 0.43 |
| PA4488 |  | conserved hypothetical protein | 0.46 |
| PA4489 |  | conserved hypothetical protein | 0.35 |
| PA4490 |  | conserved hypothetical protein | 0.43 |
| PA4491 | *pufY ;yfaA* | conserved hypothetical protein | 0.46 |
| PA4494 |  | probable two-component sensor | 0.49 |
| PA4496 |  | probable binding protein component of ABC transporter | 2.95 |
| PA4500 |  | probable binding protein component of ABC transporter | 4.42 |
| PA4501 | *opdD* | Glycine-glutamate dipeptide porin OpdP | 3.78 |
| PA4502 |  | probable binding protein component of ABC transporter | 3.27 |
| PA4507 |  | hypothetical protein | 7.79 |
| PA4517 |  | conserved hypothetical protein | 0.33 |
| PA4519 | *speC* | ornithine decarboxylase | 0.23 |
| PA4535 |  | hypothetical protein | 2.06 |
| PA4570 |  | hypothetical protein | 17.86 |
| PA4572 | *fklB* | peptidyl-prolyl cis-trans isomerase FklB | 0.45 |
| PA4573 |  | hypothetical protein | 4.04 |
| PA4574 | *yqhA* | conserved hypothetical protein | 0.37 |
| PA4587 | *ccpR* | cytochrome c551 peroxidase precursor | 0.28 |
| PA4601 | *morA* | motility regulator | 0.44 |
| PA4602 | *glyA3* | serine hydroxymethyltransferase | 0.20 |
| PA4606 | *cstA* | conserved hypothetical protein | 2.65 |
| PA4607 |  | hypothetical protein | 3.35 |
| PA4608 |  | hypothetical protein | 3.06 |
| PA4615 |  | probable oxidoreductase | 2.35 |
| PA4638 |  | hypothetical protein | 3.28 |
| PA4646 | *upp* | uracil phosphoribosyltransferase | 0.49 |
| PA4648 |  | hypothetical protein | 2.07 |
| PA4657 |  | hypothetical protein | 3.01 |
| PA4662 | *murI* | glutamate racemase | 0.50 |
| PA4665 | *prfA* | peptide chain release factor 1 | 0.38 |
| PA4670 | *prs* | ribose-phosphate pyrophosphokinase | 0.37 |
| PA4672 | *pth* | peptidyl-tRNA hydrolase | 0.31 |
| PA4673 | *ychF* | conserved hypothetical protein | 0.20 |
| PA4675 | *optH ;iutA* | probable TonB-dependent receptor | 2.19 |
| PA4676 | *yadF* | probable carbonic anhydrase | 0.48 |
| PA4685 |  | hypothetical protein | 0.47 |
| PA4694 | *ilvC* | ketol-acid reductoisomerase | 0.27 |
| PA4695 | *ilvH* | acetolactate synthase isozyme III small subunit | 0.32 |
| PA4696 | *ilvI* | acetolactate synthase large subunit | 0.20 |
| PA4702 |  | hypothetical protein | 2.61 |
| PA4706 | *phuV* | probable ATP-binding component of ABC transporter | 2.62 |
| PA4708 | *phuT* | Heme-transport protein, PhuT | 5.47 |
| PA4709 | *phuS* | probable hemin degrading factor | 5.67 |
| PA4710 | *phuR* | Haem/Haemoglobin uptake outer membrane receptor PhuR | 5.14 |
| PA4714 |  | conserved hypothetical protein | 2.50 |
| PA4720 | *trmA* | tRNA (uracil-5-)-methyltransferase | 0.50 |
| PA4727 | *pcnB* | poly(A) polymerase | 0.44 |
| PA4730 | *panC* | pantoate--beta-alanine ligase | 0.50 |
| PA4731 | *panD* | aspartate 1-decarboxylase precursor | 0.32 |
| PA4733 | *acsB* | acetyl-coenzyme A synthetase | 3.80 |
| PA4738 | *yjbJ* | conserved hypothetical protein | 3.03 |
| PA4740 | *pnp* | polyribonucleotide nucleotidyltransferase | 0.43 |
| PA4741 | *rpsO* | 30S ribosomal protein S15 | 0.50 |
| PA4742 | *truB* | tRNA pseudouridine 55 synthase | 0.43 |
| PA4743 | *rbfA* | ribosome-binding factor A | 0.27 |
| PA4744 | *infB* | translation initiation factor IF-2 | 0.43 |
| PA4746 | *yhbC* | conserved hypothetical protein | 0.49 |
| PA4753 | *yhbY* | conserved hypothetical protein | 0.34 |
| PA4756 | *carB* | carbamoylphosphate synthetase large subunit | 0.43 |
| PA4757 | *yeaS* | conserved hypothetical protein | 0.30 |
| PA4758 | *carA* | carbamoyl-phosphate synthase small chain | 0.29 |
| PA4761 | *dnaK* | DnaK protein | 2.58 |
| PA4767 | *yfjG* | conserved hypothetical protein | 2.12 |
| PA4770 | *lldP* | L-lactate permease | 2.54 |
| PA4809 | *fdhE* | FdhE protein | 0.49 |
| PA4810 | *fdnI* | nitrate-inducible formate dehydrogenase, gamma subunit | 0.44 |
| PA4811 | *fdnH* | nitrate-inducible formate dehydrogenase, beta subunit | 0.41 |
| PA4812 | *fdnG* | formate dehydrogenase-O, major subunit | 0.28 |
| PA4817 |  | hypothetical protein | 0.40 |
| PA4833 |  | conserved hypothetical protein | 2.83 |
| PA4839 | *speA* | biosynthetic arginine decarboxylase | 0.46 |
| PA4840 | *yciH* | conserved hypothetical protein | 0.41 |
| PA4844 |  | probable chemotaxis transducer | 0.18 |
| PA4847 | *accB, fabE* | biotin carboxyl carrier protein (BCCP) | 0.39 |
| PA4848 | *accC* | biotin carboxylase | 0.43 |
| PA4852 | *yhdG* | conserved hypothetical protein | 0.42 |
| PA4853 | *fis* | DNA-binding protein Fis | 0.32 |
| PA4854 | *purH* | phosphoribosylaminoimidazolecarboxamide formyltransferase | 0.33 |
| PA4855 | *purD* | phosphoribosylamine--glycine ligase | 0.28 |
| PA4876 | *osmE* | osmotically inducible lipoprotein OsmE | 2.08 |
| PA4877 |  | hypothetical protein | 4.05 |
| PA4888 | *desB* | acyl-CoA delta-9-desaturase, DesB | 0.40 |
| PA4895 |  | probable transmembrane sensor | 4.77 |
| PA4896 |  | probable sigma-70 factor, ECF subfamily | 10.34 |
| PA4907 | *ydfG* | probable short-chain dehydrogenase | 0.37 |
| PA4915 |  | probable chemotaxis transducer | 2.31 |
| PA4928 | *ygiR; ygiQ ;* | conserved hypothetical protein | 0.37 |
| PA4938 | *purA* | adenylosuccinate synthetase | 0.45 |
| PA4940 | *yjeT* | conserved hypothetical protein | 0.43 |
| PA4973 | *thiC* | thiamin biosynthesis protein ThiC | 2.80 |
| PA5015 | *aceE* | pyruvate dehydrogenase complex component E1 | 3.17 |
| PA5016 | *aceB* | pyruvate dehydrogenase complex component E2 | 2.42 |
| PA5017 |  | conserved hypothetical protein | 0.43 |
| PA5024 | *ytnM* | conserved hypothetical protein | 0.27 |
| PA5025 | *metY* | homocysteine synthase | 2.15 |
| PA5030 | *ynfM* | probable major facilitator superfamily (MFS) transporter | 0.42 |
| PA5035 | *gltD* | glutamate synthase small chain | 0.22 |
| PA5036 | *aspB* | glutamate synthase large chain precursor | 0.26 |
| PA5046 |  | malic enzyme | 0.41 |
| PA5048 |  | probable nuclease | 0.49 |
| PA5060 | *phaF* | polyhydroxyalkanoate synthesis protein PhaF | 2.98 |
| PA5072 |  | probable chemotaxis transducer | 0.28 |
| PA5075 |  | probable permease of ABC transporter | 0.50 |
| PA5094 |  | probable ATP-binding component of ABC transporter | 2.01 |
| PA5099 |  | probable transporter | 2.31 |
| PA5100 | *hutU* | urocanase | 4.56 |
| PA5112 | *estA* | esterase EstA | 2.40 |
| PA5117 | *typA* | regulatory protein TypA | 0.15 |
| PA5118 | *thiI* | thiazole biosynthesis protein ThiI | 0.30 |
| PA5119 | *glnA* | glutamine synthetase | 0.35 |
| PA5150 |  | probable short-chain dehydrogenase | 3.68 |
| PA5152 |  | probable ATP-binding component of ABC transporter | 2.46 |
| PA5153 |  | probable periplasmic binding protein | 3.34 |
| PA5157 | *marR* | probable transcriptional regulator | 0.34 |
| PA5158 | *opmG* | probable outer membrane protein precursor | 0.45 |
| PA5159 | *emrA; pmrA* | multidrug resistance protein | 0.35 |
| PA5160 | *emrB; pmrB* | drug efflux transporter | 0.50 |
| PA5174 |  | probable beta-ketoacyl synthase | 0.15 |
| PA5183 |  | hypothetical protein | 0.50 |
| PA5187 |  | probable acyl-CoA dehydrogenase | 2.30 |
| PA5191 |  | hypothetical protein | 2.12 |
| PA5217 |  | probable binding protein component of ABC iron transporter | 4.28 |
| PA5239 | *rho* | transcription termination factor Rho | 0.37 |
| PA5248 |  | hypothetical protein | 0.23 |
| PA5300 | *cycB* | cytochrome c5 | 0.41 |
| PA5312 | *kauB* | probable aldehyde dehydrogenase | 5.65 |
| PA5313 | *paaT* | probable pyridoxal-dependent aminotransferase | 2.65 |
| PA5315 | *rpmG* | 50S ribosomal protein L33 | 0.43 |
| PA5322 | *algC* | phosphomannomutase AlgC | 0.44 |
| PA5336 | *gmk* | guanylate kinase | 0.43 |
| PA5348 |  | probable DNA-binding protein | 2.28 |
| PA5352 | *glcG* | conserved hypothetical protein | 5.19 |
| PA5353 | *glcF* | glycolate oxidase subunit GlcF | 4.56 |
| PA5354 | *glcE* | glycolate oxidase subunit GlcE | 4.37 |
| PA5355 | *glcD* | glycolate oxidase subunit GlcD | 4.89 |
| PA5359 |  | hypothetical protein | 2.81 |
| PA5360 | *phoB* | two-component response regulator PhoB | 0.03 |
| PA5361 | *phoR* | two-component sensor PhoR | 0.05 |
| PA5362 |  | conserved hypothetical protein | 0.37 |
| PA5365 | *phoU* | phosphate uptake regulatory protein PhoU | 0.08 |
| PA5366 | *pstB* | ATP-binding component of ABC phosphate transporter | 0.04 |
| PA5367 | *pstA* | membrane protein component of ABC phosphate transporter | 0.04 |
| PA5368 | *pstC* | membrane protein component of ABC phosphate transporter | 0.04 |
| PA5369 | *pstS* | phosphate ABC transporter, periplasmic phosphate-binding protein, PstS | 0.03 |
| PA5380 |  | probable transcriptional regulator | 2.01 |
| PA5409 |  | hypothetical protein | 2.21 |
| PA5425 | *purK* | phosphoribosylaminoimidazole carboxylase | 0.36 |
| PA5426 | *purE* | phosphoribosylaminoimidazole carboxylase, catalytic subunit | 0.33 |
| PA5429 | *aspA* | aspartate ammonia-lyase | 5.15 |
| PA5435 | *oadA* | probable transcarboxylase subunit | 3.44 |
| PA5436 |  | probable biotin carboxylase subunit of a transcarboxylase | 3.94 |
| PA5445 | *pseA* | probable coenzyme A transferase | 2.18 |
| PA5446 |  | hypothetical protein | 0.06 |
| PA5450 | *wzt* | ABC subunit of A-band LPS efflux transporter | 2.05 |
| PA5453 | *gmd* | GDP-mannose 4,6-dehydratase | 2.77 |
| PA5454 | *rmd* | oxidoreductase Rmd | 2.72 |
| PA5460 |  | hypothetical protein | 0.14 |
| PA5463 |  | hypothetical protein | 0.36 |
| PA5464 |  | hypothetical protein | 0.49 |
| PA5468 |  | probable citrate transporter | 2.72 |
| PA5469 |  | conserved hypothetical protein | 3.17 |
| PA5482 |  | hypothetical protein | 2.17 |
| PA5490 | *cc4* | cytochrome c4 precursor | 0.40 |
| PA5491 |  | probable cytochrome | 0.46 |
| PA5505 |  | probable TonB-dependent receptor | 0.44 |
| PA5521 |  | probable short-chain dehydrogenase | 2.38 |
| PA5522 |  | probable glutamine synthetase | 2.10 |
| PA5523 |  | probable aminotransferase | 2.50 |
| PA5529 |  | probable sodium/proton antiporter | 2.05 |
| PA5530 |  | probable MFS dicarboxylate transporter | 0.35 |
| PA5531 | *tonB* | TonB protein | 2.13 |
| PA5545 |  | conserved hypothetical protein | 2.65 |
| PA5546 |  | conserved hypothetical protein | 2.00 |
| PA5549 | *glmS* | glucosamine--fructose-6-phosphate aminotransferase | 0.16 |
| PA5550 | *glmR* | GlmR transcriptional regulator | 0.28 |
| PA5553 | *atpC* | ATP synthase epsilon chain | 0.42 |
| PA5554 | *atpD* | ATP synthase beta chain | 0.44 |
| PA5561 | *atpI* | ATP synthase protein I | 0.43 |
| PA5564 | *gidB* | glucose inhibited division protein B | 0.36 |
| PA5565 | *gidA* | glucose-inhibited division protein A | 0.46 |
| PA5568 | *yidC* | conserved hypothetical protein | 0.45 |
