# Supplementary material for: Regulatory and Metabolic Networks for the Adaptation of Pseudomonas aeruginosa Biofilms to Urinary Tract-Like Conditions
Source: PLoS One. 2013 Aug 13;8(8):e71845. doi: 10.1371/journal.pone.0071845 (PMC3742457; doi:10.1371/journal.pone.0071845)
Supplement: Table S2 — Differently expressed genes involved in regulation of adaptation of P. aeruginosa PAO1 to urinary tract conditions. Pairwise comparisons between late logarithmic biofilms grown anaerobically on AUM and 10-fold diluted LB supplemented with 50 mM nitrate was performed. A fold change cut-off of two and a ppde above 0.99999 was applied. (DOCX) [file pone.0071845.s003.docx]

**Table S2. Differently expressed genes involved in regulation of adaptation of *P. aeruginosa* PAO1 to urinary tract conditions.** Pairwise comparisons between late logarithmic biofilms grown anaerobically on AUM and 10-fold diluted LB supplemented with 50 mM nitrate was performed. A fold change cut-off of two and a ppde above 0.99999 was applied.

| **Locus tag** | **Gene name** | **Function** | | | **Fold change** |
| --- | --- | --- | --- | --- | --- |
|  |  | **FUR regulon** | | |  |
| PA4764 | *fur* | ferric uptake regulation protein | | | 1.6 |
| PA2426 | *pvdS* | sigma factor PvdS | | | 6.6 |
| PA4227 | *pchR* | transcriptional regulator PchR | | | 4.5 |
| PA2258 | *ptxR* | transcriptional regulator PtxR | | | 1.1 |
| PA2686 | *pfeR* | two-component response regulator PfeR | | | 3.1 |
| PA0471 | *fiuR* | probable transmembrane sensor | | | 4.2 |
| PA0472 | *fiuI* | probable sigma-70 factor. ECF subfamily | | | 4.3 |
| PA1300 |  | probable sigma-70 factor. ECF subfamily | | | 11.7 |
| PA1301 |  | probable transmembrane sensor | | | 10.1 |
| PA1911 | *femR* | probable transmembrane sensor | | | 6.8 |
| PA1912 | *femI* | probable sigma-70 factor. ECF subfamily | | | 9.9 |
| PA2093 |  | probable sigma-70 factor. ECF subfamily | | | 6.3 |
| PA2094 |  | probable transmembrane sensor | | | 5.7 |
| PA2387 | *fpvI* | probable sigma-70 factor. ECF subfamily | | | 1.4 |
| PA2388 | *fpvR* | probable transmembrane sensor | | | 1.3 |
| PA2467 | *foxR* | Anti-sigma factor FoxR | | | 3.3 |
| PA2468 | *foxI* | ECF sigma factor FoxI | | | 4.3 |
| PA3409 |  | probable transmembrane sensor | | | 3.7 |
| PA3410 |  | probable sigma-70 factor. ECF subfamily | | | 8.4 |
| PA3899 | *fecI* | probable sigma-70 factor. ECF subfamily | | | 7.1 |
| PA3900 | *fecR* | probable transmembrane sensor | | | 3.2 |
| PA4895 |  | probable transmembrane sensor | | | 4.8 |
| PA4896 |  | probable sigma-70 factor. ECF subfamily | | | 10.3 |
| PA2385 | *pvdQ* | PvdQ | | | 6.7 |
| PA2386 | *pvdA* | L-ornithine N5-oxygenase | | | 53.5 |
| PA2389 |  | conserved hypothetical protein | | | 6.1 |
| PA2390 |  | probable ATP-binding ABC transporter | | | 3.4 |
| PA2391 | *opmQ* | probable outer membrane protein precursor | | | 2.7 |
| PA2392 | *pvdP* | PvdP | | | 12.2 |
| PA2393 |  | probable dipeptidase precursor | | | 21.5 |
| PA2394 | *pvdN* | PvdN | | | 17.5 |
| PA2395 | *pvdO* | PvdO | | | 8.8 |
| PA2396 | *pvdF* | pyoverdine synthetase F | | | 12.4 |
| PA2397 | *pvdE* | pyoverdine biosynthesis protein PvdE | | | 14.8 |
| PA2398 | *fpvA* | ferripyoverdine receptor | | | 27.6 |
| PA2399 | *pvdD* | pyoverdine synthetase D | | | 7.2 |
| PA2400 | *pvdJ* | PvdJ | | | 8.1 |
| PA2401 | *pvdI* | PvdI | | | 6.6 |
| PA2402 |  | probable non-ribosomal peptide synthetase | | | 11.3 |
| PA2403 |  | hypothetical protein | | | 15.6 |
| PA2404 |  | hypothetical protein | | | 12.2 |
| PA2405 |  | hypothetical protein | | | 22.8 |
| PA2406 |  | hypothetical protein | | | 9.5 |
| PA2407 |  | probable adhesion protein | | | 6.4 |
| PA2408 |  | ATP-binding component of ABC transporter | | | 4.6 |
| PA2409 |  | probable permease of ABC transporter | | | 2.9 |
| PA2410 |  | hypothetical protein | | | 4.8 |
| PA2411 |  | probable thioesterase | | | 42.8 |
| PA2412 |  | conserved hypothetical protein | | | 70.2 |
| PA2413 | *pvdH* | diaminobutyrate-2-ketoglutarate 4-aminotransferase | | | 33.3 |
| PA2424 | *pvdL* | PvdL | | | 13.3 |
| PA2425 | *pvdG* | PvdG | | | 9.0 |
| PA2254 | *pvcA* | pyoverdine biosynthesis protein PvcA | | | 1.0 |
| PA2255 | *pvcB* | pyoverdine biosynthesis protein PvcB | | | 0.9 |
| PA2256 | *pvcC* | pyoverdine biosynthesis protein PvcC | | | 1.0 |
| PA2257 | *pvcD* | pyoverdine biosynthesis protein PvcD | | | 1.0 |
| PA0707 | *toxR* | transcriptional regulator ToxR | | | 7.4 |
| PA1134 |  | hypothetical protein | | | 2.7 |
| PA2531 |  | probable aminotransferase | | | 3.4 |
| PA2451 |  | hypothetical protein | | | 3.1 |
| PA4221 | *fptA* | Fe(III)-pyochelin outer membrane receptor precursor | | | 3.2 |
| PA4220 | *fptB* | hypothetical protein | | | 4.0 |
| PA4222 | *pchI* | ATP-binding component of ABC transporter | | | 7.2 |
| PA4223 | *pchH* | ATP-binding component of ABC transporter | | | 6.3 |
| PA4224 | *pchG* | pyochelin biosynthetic protein PchG | | | 7.1 |
| PA4225 | *pchF* | pyochelin synthetase | | | 8.9 |
| PA4226 | *pchE* | dihydroaeruginoic acid synthetase | | | 8.1 |
| PA4227 | *pchR* | transcriptional regulator PchR | | | 4.5 |
| PA4228 | *pchD* | pyochelin biosynthesis protein PchD | | | 3.6 |
| PA4229 | *pchC* | pyochelin biosynthetic protein PchC | | | 3.7 |
| PA4230 | *pchB* | salicylate biosynthesis protein PchB | | | 5.2 |
| PA4231 | *pchA* | salicylate biosynthesis isochorismate synthase | | | 3.9 |
| PA4467 |  | hypothetical protein | | | 12.3 |
| PA4468 | *sodM* | sodA ; superoxide dismutase | | | 19.8 |
| PA4469 |  | hypothetical protein | | | 22.1 |
| PA4470 | *fumC1* | fumarate hydratase | | | 18.9 |
| PA4471 | *fagA* | hypothetical protein | | | 18.9 |
| PA2688 | *pfeA* | Ferric enterobactin receptor. outer membrane protein | | | 2.5 |
| PA4705 | *phuW* | hypothetical protein | | | 1.5 |
| PA4706 | *phuV* | ATP-binding component of ABC transporter | | | 2.6 |
| PA4707 | *phuU* | probable permease of ABC transporter | | | 1.9 |
| PA4708 | *phuT* | Heme-transport protein. PhuT | | | 5.5 |
| PA4709 | *phuS* | probable hemin degrading factor | | | 5.6 |
| PA4710 | *phuR* | Haem/Haemoglobin uptake outer membrane receptor | | | 5.1 |
| PA4514 | *piuA* | probable outer membrane receptor for iron transport | | | 0.5 |
| PA4168 | *fpvB* | ferric pyoverdine receptor FpvB | | | 5.0 |
| PA2466 | *foxA* | Ferrioxamine receptor FoxA | | | 2.0 |
| PA3405 | *hasE* | metalloprotease secretion protein | | | 2.4 |
| PA3406 | *hasD* | transport protein HasD | | | 3.2 |
| PA3407 | *hasAp* | heme acquisition protein HasAp | | | 64.0 |
| PA3408 | *hasR* | Haem uptake outer membrane receptor HasR precursor | | | 10.00 |
| PA3901 | *fecA* | Fe(III) dicitrate transport protein FecA | | | 17.1 |
| PA0929 | *pirR* | two-component response regulator | | | 3.6 |
| PA0930 | *pirS* | two-component sensor | | | 1.9 |
| PA0931 | *pirA* | ferric enterobactin receptor PirA | | | 2.4 |
|  |  | **Quorum sensing regulon** | | |  |
| PA3476 | *rhlI* | autoinducer synthesis protein RhlI | | | 1.9 |
| PA3477 | *rhlR* | transcriptional regulator RhlR | | | 1.1 |
| PA3478 | *rhlB* | rhamnosyltransferase chain B | | | 1.7 |
| PA3479 | *rhlA* | rhamnosyltransferase chain A | | | 2.8 |
| PA2570 | *lecA* | galactophilic lectin LecA | | | 1.8 |
| PA3361 | *lecB* | fucose-binding lectin LecB | | | 2.3 |
| PA4209 | *phzM* | phenazine-specific methyltransferase | | | 1.4 |
| PA4210 | *phzA1* | phenazine biosynthesis protein | | | 0.9 |
| PA4211 | *phzB1* | phenazine biosynthesis protein | | | 1.1 |
| PA4217 | *phzS* | flavin-containing monooxygenase | | | 0.8 |
| PA1901 | *phzC2* | phenazine biosynthesis protein PhzC | | | 1.4 |
| PA1902 | *phzD2* | phenazine biosynthesis protein PhzD | | | 1.1 |
| PA1903 | *phzE2* | phenazine biosynthesis protein PhzE | | | 0.9 |
| PA1904 | *phzF2* | probable phenazine biosynthesis protein | | | 1.1 |
| PA1905 | *phzG2* | probable pyridoxamine 5'-phosphate oxidase | | | 1.0 |
| PA1430 | *lasR* | transcriptional regulator LasR | | | 1.2 |
| PA1432 | *lasI* | autoinducer synthesis protein LasI | | | 1.5 |
| PA1871 | *lasA* | LasA protease precursor | | | 1.8 |
| PA2193 | *hcnA* | hydrogen cyanide synthase HcnA | | | 0.5 |
| PA2194 | *hcnB* | hydrogen cyanide synthase HcnB | | | 0.4 |
| PA2195 | *hcnC* | hydrogen cyanide synthase HcnC | | | 0.5 |
| PA3724 | *lasB* | elastase LasB | | | 4.8 |
| PA2862 | *lipA* | lactonizing lipase precursor | | | 8.8 |
| PA2863 | *lipH* | lipase modulator protein | | | 2.1 |
| PA1245 | *aprX* | hypothetical protein | | | 9.1 |
| PA1246 | *aprD* | alkaline protease secretion protein AprD | | | 3.4 |
| PA1247 | *aprE* | alkaline protease secretion protein AprE | | | 3.4 |
| PA1248 | *aprF* | Alkaline protease secretion protein AprF | | | 2.7 |
| PA1249 | *aprA* | alkaline metalloproteinase precursor AprA | | | 24.7 |
| PA1003 | *pqsR* | Transcriptional regulator MvfR | | | 6.1 |
| PA0996 | *pqsA* | probable coenzyme A ligase | | | 2.3 |
| PA0997 | *pqsB* | beta-keto-acyl-acyl-carrier protein synthase | | | 2.1 |
| PA0998 | *pqsC* | beta-keto-acyl-acyl-carrier protein synthase | | | 2.1 |
| PA0999 | *pqsD* | 3-oxoacyl-[acyl-carrier-protein] synthase III | | | 2.0 |
| PA1000 | *pqsE* | Quinolone signal response protein | | | 1.5 |
| PA1001 | *phnA* | anthranilate synthase component I | | | 1.7 |
| PA1002 | *phnB* | anthranilate synthase component II | | | 1.8 |
|  |  | | **ANR regulon** | |  |
| PA1544 | *anr* | | transcriptional regulator Anr | | 0.8 |
| PA0527 | *dnr* | | transcriptional regulator Dnr | | 0.6 |
| PA3878 | *narX* | | two-component sensor NarX | | 2.7 |
| PA3879 | *narL* | | two-component response regulator NarL | | 2.5 |
| PA3872 | *narI* | | respiratory nitrate reductase gamma chain | | 0.2 |
| PA3873 | *narJ* | | respiratory nitrate reductase delta chain | | 0.2 |
| PA3874 | *narH* | | respiratory nitrate reductase beta chain | | 0.1 |
| PA3875 | *narG* | | respiratory nitrate reductase alpha chain | | 0.2 |
| PA3876 | *narK2* | | nitrite extrusion protein 2 | | 0.5 |
| PA3877 | *narK1* | | nitrite extrusion protein 1 | | 0.2 |
| PA3870 | *moaA1* | | molybdopterin biosynthetic protein A1 | | 0.6 |
| PA4666 | *hemA* | | delta-aminolevulinic synthase | | 0.8 |
| PA3871 | *nifM* | | peptidyl-prolyl cis-trans isomerase. PpiC-type | | 0.3 |
| PA5170 | *arcD* | | arginine/ornithine antiporter | | 1.2 |
| PA5171 | *arcA* | | arginine deiminase | | 1.2 |
| PA5172 | *arcB* | | ornithine carbamoyltransferase. catabolic | | 1.6 |
| PA5173 | *arcC* | | carbamate kinase | | 1.0 |
| PA0835 | *pta* | | phosphate acetyltransferase | | 1.0 |
| PA0836 | *ackA* | | acetate kinase | | 1.2 |
| PA3929 | *cioB* | | cyanide insensitive terminal oxidase | | 0.8 |
| PA3930 | *cioA* | | cyanide insensitive terminal oxidase | | 0.8 |
| PA1553 | *cooO/fixO* | | probable cytochrome oxidase subunit (cbb3-type) | | 0.5 |
| PA1554 | *cooN/fixN* | | probable cytochrome oxidase subunit (cbb3-type) | | 0.6 |
| PA1555 | *ccoP/fixP* | | probable cytochrome c | | 0.4 |
| PA1556 | *cooO/fixO* | | probable cytochrome c oxidase subunit | | 0.4 |
| PA1557 | *cooQ/fixQ* | | probable cytochrome oxidase subunit (cbb3-type) | | 0.5 |
| PA0509 | *nirN* | | probable c-type cytochrome | | 0.3 |
| PA0510 | *nirE* | | probable uroporphyrin-III c-methyltransferase | | 0.2 |
| PA0511 | *nirJ* | | heme d1 biosynthesis protein NirJ | | 0.3 |
| PA0512 | *nirH* | | conserved hypothetical protein | | 0.3 |
| PA0513 | *nirG* | | probable transcriptional regulator | | 0.2 |
| PA0514 | *nirL* | | heme d1 biosynthesis protein NirL | | 0.3 |
| PA0515 | *nirD* | | probable transcriptional regulator | | 0.2 |
| PA0516 | *nirF* | | heme d1 biosynthesis protein NirF | | 0.2 |
| PA0517 | *nirC* | | probable c-type cytochrome precursor | | 0.1 |
| PA0518 | *nirM* | | cytochrome c-551 precursor | | 0.1 |
| PA0519 | *nirS* | | nitrite reductase precursor | | 0.2 |
| PA0520 | *nirQ* | | regulatory protein NirQ | | 0.4 |
| PA0521 | *nirO* | | probable cytochrome c oxidase subunit | | 0.3 |
| PA0522 | *nirP* | | hypothetical protein | | 0.7 |
| PA0523 | *norC* | | nitric-oxide reductase subunit C | | 0.1 |
| PA0524 | *norB* | | nitric-oxide reductase subunit B | | 0.1 |
| PA0525 | *norD* | | probable dinitrification protein NorD | | 0.2 |
| PA0526 |  | | hypothetical protein | | 0.4 |
| PA3391 | *nosR* | | regulatory protein NosR | | 0.2 |
| PA3392 | *nosZ* | | nitrous-oxide reductase precursor | | 0.1 |
| PA3393 | *nosD* | | NosD protein | | 0.2 |
| PA3394 | *nosF* | | NosF protein | | 0.2 |
| PA3395 | *nosY* | | NosY protein | | 0.3 |
| PA3396 | *nosL* | | NosL protein | | 0.4 |
|  |  | | | **PhoBR regulon** |  |
| PA5360 | *phoB* | | | two-component response regulator PhoB | 0.03 |
| PA5361 | *phoR* | | | two-component sensor PhoR | 0.1 |
| PA5362 |  | | | conserved hypothetical protein | 0.4 |
| PA5363 |  | | | hypothetical protein | 1.4 |
| PA5364 |  | | | probable two-component response regulator | 0.7 |
| PA5365 | *phoU* | | | phosphate uptake regulatory protein PhoU | 0.1 |
| PA5366 | *pstB* | | | ATP-binding component of phosphate transporter | 0.04 |
| PA5367 | *pstA* | | | membrane protein of phosphate transporter | 0.04 |
| PA5368 | *pstC* | | | membrane protein of phosphate transporter | 0.04 |
| PA5369 | *pstS* | | | periplasmic phosphate-binding protein PstS | 0.03 |
| PA3375 | *phnL* | | | probable ATP-binding component of transporter | 0.3 |
| PA3376 | *phnK* | | | probable ATP-binding component of transporter | 0.3 |
| PA3377 | *phnJ* | | | conserved hypothetical protein | 0.1 |
| PA3378 | *phnI* | | | conserved hypothetical protein | 0.1 |
| PA3379 | *phnH* | | | conserved hypothetical protein | 0.2 |
| PA3380 | *phnG* | | | conserved hypothetical protein | 0.1 |
| PA3381 | *phnF* | | | probable transcriptional regulator | 0.1 |
| PA3382 | *phnE* | | | phosphonate transport protein PhnE | 0.2 |
| PA3383 | *phnD* | | | binding protein of phosphonate transporter | 0.05 |
| PA3384 | *phnC* | | | ATP-binding component of phosphonate transporter | 0.2 |
| PA3296 | *phoA* | | | alkaline phosphatase | 0.05 |
| PA3319 | *plcN* | | | non-hemolytic phospholipase C precursor | 0.2 |
|  |  | | | **FleQ regulon** |  |
| PA1097 | *fleQ* | | | transcriptional regulator FleQ | 1.2 |
| PA1077 | *flgB* | | | flagellar basal-body rod protein FlgB | 0.2 |
| PA1078 | *flgC* | | | flagellar basal-body rod protein FlgC | 0.2 |
| PA1079 | *flgD* | | | flagellar basal-body rod modification protein FlgD | 0.1 |
| PA1080 | *flgE* | | | flagellar hook protein FlgE | 0.2 |
| PA1081 | *flgF* | | | flagellar basal-body rod protein FlgF | 0.2 |
| PA1082 | *flgG* | | | flagellar basal-body rod protein FlgG | 0.3 |
| PA1083 | *flgH* | | | flagellar L-ring protein precursor FlgH | 0.3 |
| PA1084 | *flgI* | | | flagellar P-ring protein precursor FlgI | 0.3 |
| PA1085 | *flgJ* | | | flagellar protein FlgJ | 0.3 |
| PA1086 | *flgK* | | | flagellar hook-associated protein 1 FlgK | 0.2 |
| PA1087 | *flgL* | | | flagellar hook-associated protein type 3 FlgL | 0.2 |
| PA1088 |  | | | hypothetical protein | 0.3 |
| PA1089 |  | | | conserved hypothetical protein | 0.3 |
| PA1090 |  | | | hypothetical protein | 0.3 |
| PA1091 | *fgtA* | | | flagellar glycosyl transferase. FgtA | 0.3 |
| PA1092 | *fliC* | | | flagellin type B | 0.5 |
| PA1093 | *flaG* | | | hypothetical protein | 0.4 |
| PA1094 | *fliD* | | | flagellar capping protein FliD | 0.5 |
| PA1095 | *fliS* | | | hypothetical protein | 0.3 |
| PA1096 |  | | | hypothetical protein | 0.2 |
| PA1097 | *fleQ* | | | transcriptional regulator FleQ | 1.2 |
| PA1098 | *fleS* | | | two-component sensor | 0.4 |
| PA1099 | *fleR* | | | two-component response regulator | 0.4 |
| PA1100 | *fliE* | | | flagellar hook-basal body complex protein FliE | 0.4 |
| PA1101 | *fliF* | | | Flagella M-ring outer membrane protein precursor | 0.4 |
| PA1102 | *fliG* | | | flagellar motor switch protein FliG | 0.7 |
| PA1103 | *fliH* | | | probable flagellar assembly protein | 0.6 |
| PA1104 | *fliI* | | | flagellum-specific ATP synthase FliI | 0.6 |
| PA1105 | *fliJ* | | | flagellar protein FliJ | 0.5 |
|  |  | | | **Vfr regulon** |  |
| PA0652 | *vfr* | | | transcriptional regulator Vfr, cyclic AMP receptor | 1.3 |
| PA1148 | *toxA* | | | exotoxin A precursor | 1.0 |
| PA0707 | *regA/toxR* | | | transcriptional regulator ToxR | 7.4 |
| PA3477 | *rhlR* | | | transcriptional regulator RhlR | 1.1 |
| PA1430 | *lasR* | | | transcriptional regulator LasR | 1.2 |
| PA5261 | *algR* | | | alginate biosynthesis regulatory protein AlgR | 1.7 |
| PA5262 | *algZ* | | | alginate biosynthesis protein AlgZ | 1.4 |
| PA1097 | *fleQ* | | | transcriptional regulator FleQ | 1.1 |
