# Supplementary material for: Regulatory and Metabolic Networks for the Adaptation of Pseudomonas aeruginosa Biofilms to Urinary Tract-Like Conditions
Source: PLoS One. 2013 Aug 13;8(8):e71845. doi: 10.1371/journal.pone.0071845 (PMC3742457; doi:10.1371/journal.pone.0071845)
Supplement: Table S3 — Differently expressed genes of P. aeruginosa PAO1 involved in central metabolism. Pairwise comparison between AUM-grown and 10-fold diluted LB-grown biofilms were performed. A fold change cut-off of two and a ppde above 0.99999 was applied. (DOCX) [file pone.0071845.s004.docx]

**Table S3. Differently expressed genes of *P. aeruginosa* PAO1 involved in central metabolism.** Pairwise comparison between AUM-grown and 10-fold diluted LB-grown biofilms were performed. A fold change cut-off of two and a ppde above 0.99999 was applied.

| **Locus tag** | **Gene name** | **Function** | **Fold change** |
| --- | --- | --- | --- |
|  |  | **Entner-Doudoroff-pathway/Gluconeogenese** |  |
| PA4732 | *pgi* | Glucose-6-phosphate isomerase | 1.2 |
| PA3183 | *zwf* | Glucose-6-phosphate 1-dehydrogenase | 1.2 |
| PA3182 | *pgl* | 6-phosphogluconolactonase | 1.1 |
| PA3184 | *edd* | Phosphogluconate dehydratase | 1.0 |
| PA3131 | *edaB* | Probable aldolase | 1.4 |
| PA3181 | *edaA* | 2-keto-3-deoxy-6-phosphogluconate aldolase | 1.1 |
| PA3195 | *gapA* | Glyceraldehyde 3-phosphate dehydrogenase Phosphoglycerate | 2.9 |
| PA0552 | *pgk* | kinase | 0.9 |
| PA5131 | *pgm* | Phosphoglycerate mutase | 0.7 |
| PA3635 | *eno* | Enolase | 0.6 |
| PA4329 | *pykA* | Phosphoenolpyruvate kinase | 0.8 |
| PA5436 | *pykA* | Subunit of transcarboxylase | 3.9 |
|  |  | **Citrate cycle/glyoxylate shunt** |  |
| PA3416 |  | Probable pyruvate dehydrogenase, beta subunit | 2.2 |
| PA3417 |  | Probable pyruvate dehydrogenase, alpha subunit | 2.3 |
| PA5015 | *aceE* | Pyruvate dehydrogenase | 3.2 |
| PA5016 | *aceB* | Malate synthase 1 | 2.4 |
| PA0482 | *glcB* | Malate synthase 2 | 1.5 |
| PA1376 | *aceK* | Isocitrate dehydrogenase kinase/phosphatase | 2.0 |
| PA0796 | *prpB* | Isocitrate lyase | 1.3 |
| PA2634 | *aceA* | Isocitrate lyase | 0.7 |
| PA0755 | *opdH* | Cis-aconitate porin OpdH | 9.3 |
| PA1580 | *gltA* | Citrate synthase 2 | 1.5 |
| PA0795 | *prpC* | Citrate synthase 2 | 3.1 |
| PA0794 |  | Probable aconitate hydratase | 1.2 |
| PA1562 | *acnA* | Aconitate hydratase 2 | 0.9 |
| PA1787 | *acnB* | Aconitate hydratase 3 | 0.4 |
| PA2623 | *icd* | Isocitrate dehydrogenase | 3.5 |
| PA2624 | *idh* | Isocitrate dehydrogenase | 1.2 |
| PA1585 | *sucA* | 2-oxoglutarate dehydrogenase (E1 subunit) | 0.9 |
| PA1586 | *sucB* | Dihydrolipoamide succinyltransferase (E2 subunit) | 0.8 |
| PA1588 | *sucC* | Succinyl-CoA synthetase beta chain | 0.8 |
| PA1589 | *sucD* | Succinyl-CoA synthetase alpha chain | 0.7 |
| PA1583 | *sdhA* | Succinate dehydrogenase (A subunit) | 0.5 |
| PA1584 | *sdhB* | Succinate dehydrogenase (B subunit) | 0.6 |
| PA1581 | *sdhC* | Succinate dehydrogenase (C subunit) | 0.6 |
| PA1582 | *sdhD* | Succinate dehydrogenase (D subunit) | 0.6 |
| PA4333 | *fumA* | Fumarase A | 0.3 |
| PA4470 | *fumC1* | Fumarase C1 | 18.9 |
| PA0854 | *fumC2* | Fumerase C2 | 0.9 |
| PA4640 | *mqoB* | Malate:quinone oxidoreductase | 1.3 |
| PA1252 |  | Probable malate dehydrogenase | 1.5 |
| PA0887 | *acsA* | Acetyl-coenzyme A synthetase | 6.4 |
| PA4733 | *acsB* | Acetyl-coenzyme A synthetase | 3.8 |
|  |  | **Glycolate degradation** |  |
| PA1498 | *pykF* | Phosphoenolpyruvate kinase | 1.2 |
| PA1499 |  | Glycerate kinase II | 1.6 |
| PA1500 | *glxR* | Probable oxidoreductase | 3.1 |
| PA1501 | *glxK* | Hydroxyl-pyruvate isomerase | 2.3 |
| PA1502 | *gcl* | Glycolate carboligase | 2.8 |
| PA5352 | *glcG* | Conserved hypothetical protein | 5.2 |
| PA5353 | *glcF* | Glycolate oxidase subunit | 4.6 |
| PA5354 | *glcE* | Glycolate oxidase subunit | 4.4 |
| PA5355 | *glcD* | Glycolate oxidase subunit | 4.9 |
