# Supplementary material for: Regulatory and Metabolic Networks for the Adaptation of Pseudomonas aeruginosa Biofilms to Urinary Tract-Like Conditions
Source: PLoS One. 2013 Aug 13;8(8):e71845. doi: 10.1371/journal.pone.0071845 (PMC3742457; doi:10.1371/journal.pone.0071845)
Supplement: Table S4 — Differently expressed genes of P. aeruginosa PAO1 involved in amino acid metabolism. Pairwise comparison between AUM-grown and 10-fold diluted LB-grown biofilms were performed. A fold change cut-off of two and a ppde above 0.99999 was applied. (DOCX) [file pone.0071845.s005.docx]

**Table S4.** Differently expressed genes of *P. aeruginosa* PAO1 involved in amino acid metabolism. Pairwise comparison between AUM-grown and 10-fold diluted LB-grown biofilms were performed. A fold change cut-off of two and a ppde above 0.99999 was applied.

| **Locus tag** | **Gene name** | **Function** | **Fold change** |
| --- | --- | --- | --- |
| PA0747 |  | Probable aldehyde dehydrogenase | 3.1 |
| PA5119 | *glnA* | Glutamine synthetase | 0.4 |
| PA0297 | *spuA* | Probable glutamine amidotransferase | 2.5 |
| PA0298 | *spuB* | Probable glutamine synthetase | 3.7 |
| PA0299 | *spuC* | Putrescine aminotransferase | 4.3 |
| PA1337 | *ansB* | Glutaminase-asparaginase | 3.2 |
| PA5429 | *aspA* | Ammonia-lyase | 5.2 |
| PA2085 |  | Probable asparagine synthetase | 10.6 |
| PA0870 | *phhC* | Aromatic amino acid aminotransferase | 4.2 |
| PA0871 | *phhB* | Pterin-4-alpha-carbinolamine dehydratase | 4.1 |
| PA0872 | *phhA* | Phenylalanine-4-hydroxylase | 2.4 |
| PA0743 |  | Probable 3-hydroxyisobutyrate dehydrogenase | 3.4 |
| PA0744 |  | Probable enoyl-CoA hydratase/isomerase | 2.8 |
| PA0745 |  | Probable enoyl-CoA hydratase/isomerase | 4.5 |
| PA0746 |  | probable acyl-CoA dehydrogenase | 2.8 |
| PA0782 | *putA* | Proline dehydrogenase PutA | 4.4 |
| PA0783 | *putP* | Sodium/proline symporter | 4.1 |
| PA0865 | *hpd* | 4-hydroxyphenylpyruvate dioxygenase | 5.4 |
| PA5304 | *dadA* | D-amino acid dehydrogenase, small subunit | 1.6 |
| PA2009 | *hmgA* | Homogentisate 1,2-dioxygenase | 3.9 |
| PA2008 | *fahA* | Fumarylacetoacetase | 3.8 |
| PA2007 | *maiA* | Maleylacetoacetate isomerase | 4.7 |
| PA1001 | *phnA* | Anthranilate synthase component I | 5.3 |
| PA1002 | *phnB* | Anthranilate synthase component II | 4.4 |
| PA2531 |  | Probable aminotransferase | 3.4 |
| PA1587 | *lpdG* | Lipoamide dehydrogenase - glycine | 0.7 |
| PA2250 | *lpdV* | Lipoamide dehydrogenase – valine | 2.2 |
| PA2247 | *bkdA1* | Oxoisovalerate dehydrogenase (alpha subunit) | 2.6 |
| PA2248 | *bkdA2* | 2-oxoisovalerate dehydrogenase (beta subunit) | 2.9 |
| PA2249 | *bkdB* | Branched-chain alpha-keto acid dehydrogenase | 2.2 |
| PA3418 | *ldh* | Leucine dehydrogenase | 3.0 |
| PA3569 | *mmsB* | 3-hydroxyisobutyrate dehydrogenase | 2.8 |
