# Supplementary material for: Regulatory and Metabolic Networks for the Adaptation of Pseudomonas aeruginosa Biofilms to Urinary Tract-Like Conditions
Source: PLoS One. 2013 Aug 13;8(8):e71845. doi: 10.1371/journal.pone.0071845 (PMC3742457; doi:10.1371/journal.pone.0071845)
Supplement: Table S5 — Proteins varying in their cellular concentration in biofilm cells of P. aeruginosa PAO1. The bacterium was grown up to the late logarithmic phase on AUM compared to 10-fold diluted LB observed via 2D gel electrophoresis and subsequently MALDI-TOF analyses. For growth under anaerobic conditions the media were supplemented with 50 mM nitrate. A fold change cutoff of 10.0 was applied. (DOCX) [file pone.0071845.s006.docx]

**Table S5. Proteins varying in their cellular concentration in biofilm cells of *P. aeruginosa* PAO1.** The bacterium was grown up to the late logarithmic phase on AUM compared to 10-fold diluted LB observed via 2D gel electrophoresis and subsequently MALDI-TOF analyses. For growth under anaerobic conditions the media were supplemented with 50 mM nitrate. A fold change cutoff of 10.0 was applied.

| **Locus tag** | **Protein name** | **Function** | **Fold Change** | |
| --- | --- | --- | --- | --- |
|  |  |  | **AUM** | **1:10 LB** |
| PA0083 |  | Hypothetical protein |  | ∞ |
| PA0459 |  | Probable ClpA/B protease ATP binding subunit |  | ∞ |
| PA0755 | OpdH | Cis-Aconitate porin | ∞ |  |
| PA0766 | MucA | Alginate biosynthesis regulator | ∞ |  |
| PA0931 | FepA | Ferric enterobactin outer membrane receptor | ∞ |  |
| PA1337 | AnsB | Asparaginase | ∞ |  |
| PA2086 |  | Probable epoxide hydrolase |  | ∞ |
| PA2396 | PvdF | Pyoverdine synthetase F | ∞ |  |
| PA2483 |  | Hypothetical protein |  | ∞ |
| PA2505 | OpdT | Tyrosine Porin | ∞ |  |
| PA2594 |  | Putative periplasmic aliphatic sulfonate-binding protein |  | ∞ |
| PA3901 | FecA | Fe(III) citrate transport portein | ∞ |  |
| PA4221 | FptA | Fe(III)-pyochelin outer membrane receptor | ∞ |  |
| PA4468 | SodM | Superoxide dismutase | ∞ |  |
| PA4470 | FumC1 | Fumarase C1 | ∞ |  |
| PA4710 | PhuR | Heme/hemeoglobin uptake outer membrane receptor PhuR precursor | ∞ |  |
| PA4848 | AccC | Pyruvate carboxylase | 12.3 |  |
| PA5103 |  | Putative ABC transporter, periplasmic substrate-binding protein |  | ∞ |
| PA5251 | AlgR | Alginate biosynthesis regulatory protein AlgR |  | ∞ |
| ∞ protein exclusively present in this condition | | | | |
