# Supplementary material for: Regulatory and Metabolic Networks for the Adaptation of Pseudomonas aeruginosa Biofilms to Urinary Tract-Like Conditions
Source: PLoS One. 2013 Aug 13;8(8):e71845. doi: 10.1371/journal.pone.0071845 (PMC3742457; doi:10.1371/journal.pone.0071845)
Supplement: Table S6 — Metabolites varying in their cellular concentration in cells of P. aeruginosa PAO1. The bacterium was grown as biofilm under anaerobic conditions up to the late logarithmic phase. Pairwise comparisons between biofilms grown with AUM supplemented with 50 mM nitrate and biofilms grown with 10-fold diluted LB supplemented with 50 mM nitrate were performed. A fold change cutoff of two and a p-value of <10−5 was applied. (DOCX) [file pone.0071845.s007.docx]

**Table S6.** **Metabolites varying in their cellular concentration in cells of *P. aeruginosa* PAO1**. The bacterium was grown as biofilm under anaerobic conditions up to the late logarithmic phase. Pairwise comparisons between biofilms grown with AUM supplemented with 50 mM nitrate and biofilms grown with 10-fold diluted LB supplemented with 50 mM nitrate were performed. A fold change cutoff of two and a *p*-value of < 10^-5^ was applied.

| **Metabolites** | **Fold change** |
| --- | --- |
| 1-Pyrroline-3-hydroxy-5-carboxylic acid | 1292.40 |
| 2-Hydroxybutanoic acid | 0.001 |
| 2-Hydroxyglutaric acid | 13.46 |
| 2-Ketoglutaric acid | 4.70 |
| 3-Phosphoglyceric acid | 21.01 |
| 4-Aminobutanoic acid | 10.48 |
| 5-Aminopentanoic acid | 29.06 |
| 5-Oxoproline | 2.96 |
| 5'-Methylthioadenosine | 0.33 |
| 6-Phosphogluconic acid | 12.34 |
| Alanine | 11.39 |
| Aspartic acid | 8.08 |
| beta-Alanine | 4.91 |
| Dihydroorotic acid | 16.64 |
| Fructose | 8.65 |
| Fumaric acid | 3.87 |
| Glucose-6-phosphoric acid | 3.07 |
| Glutamic acid | 15.51 |
| Glutamine | 2638.40 |
| Glutaric acid | 0.03 |
| Glycerol | 2.79 |
| Glycerol-3-phosphoric acid | 2.98 |
| Malic acid | 4.82 |
| Mannose | 7.21 |
| Mannose-6-phosphoric acid | 3.70 |
| N-Acetylglucosamine | 0.07 |
| N-Acetylmannosamine | 0.04 |
| O-Phospho-serine | 571.54 |
| Ornithine | 175.07 |
| Palmitic acid amide | 0.08 |
| Phosphoenolpyruvic acid | 17.20 |
| Phosphoethanolamine | 25.01 |
| Serine | 27.18 |
| Uridine | 0.28 |
